# Tree frog attachment: mechanisms, challenges, and perspectives

**DOI:** 10.1186/s12983-018-0273-x

**Published:** 2018-08-23

**Authors:** Julian K. A. Langowski, Dimitra Dodou, Marleen Kamperman, Johan L. van Leeuwen

**Affiliations:** 1 Experimental Zoology Group, Department of Animal Sciences, Wageningen University & Research, De Elst 1, Wageningen, 6708 WD The Netherlands; 2 Department of BioMechanical Engineering, Faculty of Mechanical, Maritime and Materials Engineering, Delft University of Technology, Mekelweg 2, Delft, 2628 CD The Netherlands; 3 Physical Chemistry and Soft Matter, Department of Agrotechnology and Food Sciences, Wageningen University & Research, Stippeneng 4, Wageningen, 6708 WE The Netherlands

**Keywords:** Toe pad, Attachment organ, Bioadhesion, Biotribology, Capillary adhesion, van der Waals, Drainage, Lubrication, Biomimetics, *Litoria caerulea*

## Abstract

Tree frogs have the remarkable ability to attach to smooth, rough, dry, and wet surfaces using their versatile toe pads. Tree frog attachment involves the secretion of mucus into the pad-substrate gap, requiring adaptations towards mucus drainage and pad lubrication. Here, we present an overview of tree frog attachment, with focus on (i) the morphology and material of the toe pad; (ii) the functional demands on the toe pad arising from ecology, lifestyle, and phylogenetics; (iii) experimental data of attachment performance such as adhesion and friction forces; and (iv) potential perspectives on future developments in the field. By revisiting reported data and observations, we discuss the involved mechanisms of attachment and propose new hypotheses for further research. Among others, we address the following questions: Do capillary and hydrodynamic forces explain the strong friction of the toe pads directly, or indirectly by promoting dry attachment mechanisms? If friction primarily relies on van der Waals (vdW) forces instead, how much do these forces contribute to adhesion in the wet environment tree frogs live in and what role does the mucus play? We show that both pad morphology and measured attachment performance suggest the coaction of several attachment mechanisms (e.g. capillary and hydrodynamic adhesion, mechanical interlocking, and vdW forces) with situation-dependent relative importance. Current analytical models of capillary and hydrodynamic adhesion, caused by the secreted mucus and by environmental liquids, do not capture the contributions of these mechanisms in a comprehensive and accurate way. We argue that the soft pad material and a hierarchical surface pattern on the ventral pad surface enhance the effective contact area and facilitate gap-closure by macro- to nanoscopic drainage of interstitial liquids, which may give rise to a significant contribution of vdW interactions to tree frog attachment. Increasing the comprehension of the complex mechanism of tree frog attachment contributes to a better understanding of other biological attachment systems (e.g. in geckos and insects) and is expected to stimulate the development of a wide array of bioinspired adhesive applications.

**Electronic supplementary material:**

The online version of this article (10.1186/s12983-018-0273-x) contains supplementary material, which is available to authorized users.

## Background

Strong, reversible, and repeatable grip to diverse substrates is a basic requirement for climbing animals [1]. A wide range of attachment organs fulfilling this requirement has evolved in animals such as insects [2], reptiles [3, 4], arachnids [5], and amphibians including tree [6] and torrent frogs [7]. The research on torrent frogs is relatively new and limited to a few studies [8–12], and thus this review focusses on tree frogs.

With their toe pads, tree frogs attach to a wide range of substrates, from smooth glass to rough wood [13] in both dry and wet environments [10]. Tree frogs are a polyphyletic group [14–17], but the basic morphology of their toe pads is consistent among frog families—a sign of convergent evolution [18–21]: the pads are soft (with an effective elastic modulus of ca. 20 kPa; e.g. [22]) and ventrally covered with a hierarchical, micro- to nanoscopic pattern of prismatic, epidermal cells separated by channels [23].

Several attachment mechanisms have been proposed for tree frogs’ toe pads (e.g. [6, 24, 25]). The prevailing hypothesis is that adhesion (i.e. the attachment force normal to the substrate surface) is induced by mucus that is present at the pad-substrate interface, leading to capillary and hydrodynamic forces (i.e. wet adhesion). Furthermore, intermolecular interactions (i.e. van der Waals [vdW] forces) and mechanical interlocking have been suggested to contribute to both adhesion and friction (i.e. the attachment force parallel to the substrate surface) [24]^1^.

Despite the substantial progress made in the understanding of tree frog attachment over the last centuries, several questions remain unanswered. For example, do capillary and hydrodynamic forces explain the strong friction of the toe pads directly, or indirectly by promoting dry attachment mechanisms? If friction primarily relies on vdW forces instead, how much do these forces contribute to adhesion in the wet environment tree frogs live in and what is the function of the mucus? Are there other attachment mechanisms active in the toe pads and how do these mechanisms interact? Several questions concerning the functional morphology of the attachment apparatus also remain open. Can the smooth soft toe pads of tree frogs conform closely to rough substrates to form a large area of dry contact and strong vdW forces, as described for the hairy attachment organs of geckos [26, 27] and for the soft technical adhesives inspired thereof [28–30]? Do the structures on (and in) the ventral epidermis support force generation and how are contact forces transmitted to other body parts? Do internal pad structures facilitate the spatial distribution of mechanical stresses or of energy?

We discuss these questions by revisiting evidence regarding the attachment performance of tree frogs or, when information is lacking, formulate new hypotheses for further research. First, we describe the morphology and material properties of the toe pads. Subsequently, a set of functional demands regarding adhesion and friction, which the toe pad presumably accommodates, is presented as well as the physical fundamentals of the mechanisms that have been proposed in previous research to explain tree frog attachment. Next, we discuss the observed attachment performance of tree frogs with respect to the stated questions, the functional demands, the morphological and material properties of the pad, and the physical fundamentals of attachment. Finally, we present conclusions of the reviewed knowledge available on tree frog attachment and provide perspectives for potential future developments in the field.

## Morphology and material properties of a toe pad

**Fig. 1:**
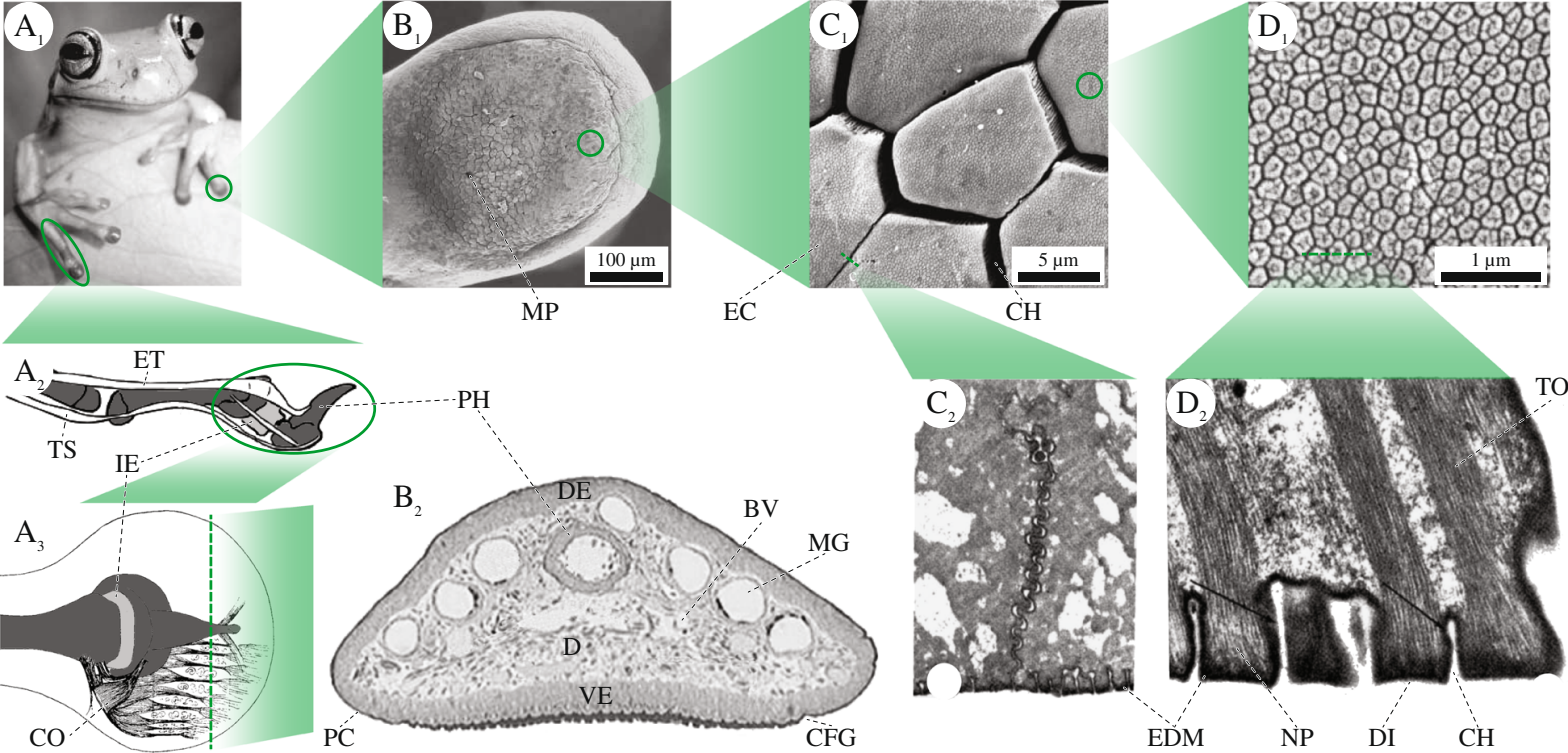
Morphology of a digit and toe pad of a hylid tree frog. **A** Macroscopic structures. (A 1) Forelimbs of *Litoria caerulea*. (A 2) Schematic lateral view of tendons, phalangi (dark grey), and the intercalary element (light grey) in a digit of *Scinax squalirostris*. (A 3) Schematic depiction of the collagen fibres in a pad of *Hyla dominicensis* in dorsal view. **B** Superficial and internal pad structures in *L. caerulea*. (B 1) SEM image of the ventral epidermis. (B 2) Transverse section through the toe of a juvenile frog. **C** Epidermal cells on the ventral surface. (C 1) SEM image of polygonal cells in *L. caerulea*. (C 2) TEM image of a tangential cross-section through the apical part of two adjacent cells in *Hyla cinerea*. **D** Fine structures of the apical surface of an epidermal cell. (D 1) High power SEM image of nanopillars and their central depressions (‘dimples’) in *L. caerulea*. (D 2) TEM image of a cross-section through a row of nanopillars in *H. cinerea* (black arrows: EDM). BV blood vessels, CFG circumferential groove, CH channel between two epidermal cells/nanopillars, CO collagen fibres, D dermis, DE dorsal epidermis, DI dimple, EC epidermal cell, EDM electron dense material, ET extensor brevis profundus tendon, IE intercalary element, MG mucus gland, MP mucus pore, NP nanopillar, PC pad curvature, PH (terminal) phalanx, TO tonofilaments, TS tendo superficialis, VE ventral epidermis. The illustrations are not to scale. A 1, B 1, C 1 and D 1 modified after [25]; A 2 modified after [66]; A 3 modified after [44]; B 2 modified after [47]; C 2 and D 1 modified after [23]. All figures printed with permission

In this section, we describe the morphology of the limbs of tree frogs from the macroscopic anatomy (Fig. 1A_1_) down to the nanoscopic features of the toe pad epidermis (Fig. 1D_2_). To get insight in where and how contact forces are generated, we categorise the morphological elements based on their potential functionality (e.g. attachment control and force transmission). Furthermore, we discuss the material properties of the pad and the secreted mucus. For open questions on the pad morphology (and for possible approaches to answer these), we refer to the final section.

### Functional morphology of limbs and toes

The tip of a tree frog’s digit consists of the terminal phalanx, dermis, and epidermis (Fig. 1B 2; [23, 31, 32]). The dermis contains connective tissue, blood vessels, lymph space, mucus glands, as well as muscle and nerve fibres (Fig. 1B 2; [23, 33, 34]). The ventral epidermis constitutes the actual toe pad [32]. The surface area *A*_p_ of single pads was reported by Linnenbach ([35]; *Hyla cinerea*, 0.82–1.21 mm^2^), Ba-Omar et al. ([36]; *Phyllomedusa trinitatis*, pad diameter *d*_p_ forelimb: 2.81 mm, *d*_p_ hindlimb: 2.47 mm), Mizuhira ([37]; two Rhacophoridae, *A*_p_ = 2.5 mm · 1.8 mm), Chakraborti et al. ([38]; *Philautus annandalii*, *d*_p_ = 1.2–1.5 mm), and Endlein et al. ([39]; *Rhacophorus dennysi*, 2.1–4.7 mm^2^). The projected surface area *A* of all pads of an individual frog scales nearly isometrically with snout-vent-length *ℓ*_SV_ (*A*∝*ℓ*_SV_^1.76−2.29^; [40–42]) and with body mass *m* (*A*∝*m*^0.68^; [43]).

#### Contact geometry

The distal portions of the toes are dilated [24, 44] and typically disc-shaped (Fig. 1B 1; [36]). The unloaded ventral toe pad surface is convex [45, 46], with a radius of curvature *R* of 0.72–1.57 mm in juveniles and 4.07–5.81 mm in adults of *Litoria caerulea* [47]. Gu et al. [48] suggested that the ball-on-flat arrangement of the curved pad on a flat substrate protects the pad from misalignment. Moreover, a curved pad might require less energy for active alignment of the pad with respect to the substrate. The ventral pad surface is divided into subunits forming a hierarchical surface pattern:

##### Macroscale

In several species, grooves following the proximal-distal axis separate the pad surface, and a circumferential groove forms the lateroterminal pad boundary between proximal (squamous) and distal (columnar) ventral epidermis (e.g. [8, 18, 20, 32, 33, 49]).

##### Microscale

**Fig. 2:**
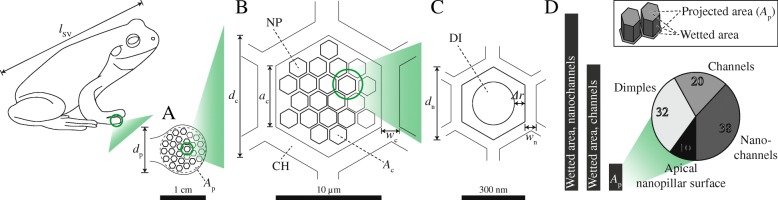
Geometrical model of the ventral toe pad epidermis in a tree frog with snout-vent-length *ℓ*_SV_. **A** The approximately circular projected ventral pad area *A*_p_ (diameter *d*_p_) is **B** covered by polygonal epidermal cells with diameter *d*_c_, edge length *a*_c_, channel width *w*_c_, and apical surface area *A*_c_. **C** Each cell accommodates polygonal nanopillars with diameter *d*_n_, channel width *w*_n_, and a distance *Δr* between dimple and nanopillar edge. **D** Enhancement (in %) of the wetted area by the micro- and nanochannels relative to *A*_p_ and composition of *A*_p_ (in %) assuming regular, hexagonal outlines of the epidermal cells and nanopillars. Inset: Definition of projected and wetted areas shown for a hexagonal pillar. CH channel, DI dimple, NP nanopillar

Prismatic cells on the ventral epidermis surface form a pattern of columnar pillars [18, 23, 32]. The apical parts of neighbouring surface cells are laterally separated by a channel network ([50, 51]; Fig. 1C 1). In *L. caerulea*, the superficial epidermal cells are skewed such that the apical cell surface is positioned more distally than the basal one [34]. The outline of the apical epidermal cell surface in *L. caerulea* and several other species is exclusively polygonal, ranging from pentagonal to octagonal (e.g. [21, 22, 52]). In *L. caerulea*, Barnes et al. [21] found 65.4% hexagonal, 19.8% pentagonal, 14.2% heptagonal, and 0.6% octagonal, non-randomly distributed cells. Chen et al. [53] reported a similar distribution with 55% of hexagonal cells, and an elongation of the cells along the proximal-distal pad-axis (aspect ratio =1.46) in *Polypedates megacephalus*. The apical cell surfaces are curved convexly [25, 50]. In *H. cinerea*, the average edge length *a*_c_ of the apically separated cells is 10.2 µm [23], the cell height *h*_c_ is 6.5 µm, and the apical cell surface *A*_c_ is 64 µm^2^ [35]. Similar values for *A*_c_ (63–172 µm^2^) and cell diameter *d*_c_ (8–14.8 µm; see Fig. 2B) were reported for a number of species [18, 19, 36, 54, 55]). Smith et al. [42] found a positive correlation between *A*_c_ and *ℓ*_SV_ (*r*=0.86; *p*=0.01; 1–2 frogs per species), which was observed neither by McAllister & Channing ([19]; 1–2 frogs per species) nor by Green ([54]; *r*=0.036; 12–17 frogs per species). Further work is required to conclude on the scaling of cell dimensions with *ℓ*_SV_. The cell density *ρ*_c_ (cells per mm^2^ toe pad area) ranges between ca. 2450 and 15700 mm ^−2^ [35, 42, 56].

The channels in between the superficial epidermal cells are 1–5 µm wide [42, 51]. In *P. megacephalus*, the channel alignment is anisotropic; the cumulative channel length is ca. 70% lower along the lateral pad axis than along the proximal-distal axis [53]. Mucus glands with large lumina are distributed in the dermis of the distal digital segment (Fig. 1B 2; [51]) and secrete mucus [57] via ducts and 7–8 µm wide pores into the epidermal channel network [58]. The spatial density and distribution pattern of the pores vary interspecifically [18, 19, 36]. A detailed analysis of the mucus gland morphology is unavailable.

##### Nanoscale

Peg-like protrusions, called nanopillars (also plaques, pegs, or microvilli), cover the apical surface of the outermost epidermal cells ([33]; Fig. 1D 1,2). Nanopillars are prismatic structures separated from each other by a nanoscopic channel network analogously to the microscopic channel network between the epidermal cells [23, 51]. For various species, nanopillar diameters (*d*_n_) of 15–800 nm were reported [18, 21–23, 37, 59]. In *L. caerulea*, the nanopillars have a (mostly) hexagonal outline with an aspect ratio of approximately 1 and a nanochannel width *w*_n_ ≪ *d*_n_ [22]. Measurement of *w*_*n*_ by atomic force microscopy (AFM) presumably underestimates the channel width (and depth; [22]). AFM and transmission electron microscopy (TEM) measurements of the nanopillars [23, 25] and cryo-scanning electron microscopy (SEM; [21]) indicated a 7.7±4.2 nm deep ‘dimple’ on the apical surface (Fig. 1D 2; [22]).

##### Geometrical model of the epidermis

Based on the dimensions of the epidermal cells reported in literature, we built a geometrical model (Fig. 2) of the epidermis to predict the increase in surface area by the cellular structures and the effective contact surface (that we assume to be formed by the apical nanopillar surfaces not covered by dimples). For calculations of the parameter values, see Additional file 1.

For an approximately circular pad with diameter *d*_p_ = 3.6 mm, we compute a projected ventral area *A*_p_ ≈ 10.2 mm^2^ covered with about 126·10^3^ epidermal cells, which agrees with the cell densities reported for real animals [35, 42]. Cells with a regular hexagonal outline (*d*_c_ = 10 µm, *h*_c_ = 10 µm, *w*_c_ = 1 µm) increase the wetted contact area (i.e. the projected ventral surface + surface of the channel walls) 4.7-fold compared to a smooth pad. Nanopillars (*d*_n_ = 300 nm, *h*_n_ = 300 nm, *w*_n_ = 100 nm according to Fig. 3 in [21], dimple diameter ≈*d*_n_−*Δr*=240 nm) cover the apical surface of every cell. The whole pad contains ca. 73·10^6^ n. This corresponds with a nanopillar density of ca. 7.1·10^6^ mm^−2^, which is in the same order of magnitude as the setae densities reported in geckos [60]. Together, the epidermal cells and nanopillars enlarge the wetted contact area 6.6-fold compared to a smooth pad. About 20% of *A*_p_ is formed by the intercellular channel network. Including the nanoscopic channel network, this fraction rises to around 58%. Dimples occupy 32% of the pad. Finally, about 10% of *A*_p_ is not covered by channels or dimples.

**Fig. 3:**
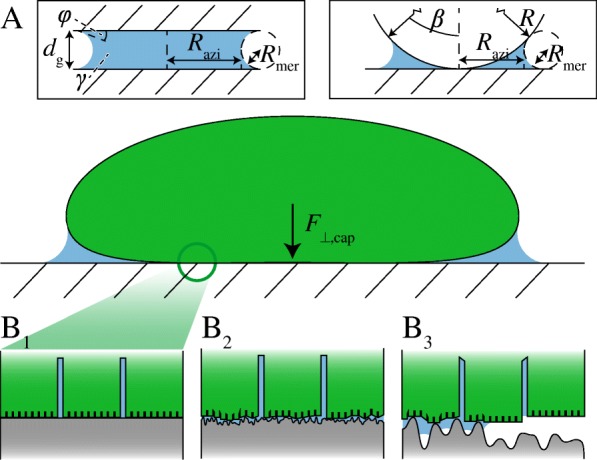
**A** Schematic representation of capillary adhesion between a toe pad (green) and a hydrophilic substrate caused by the formation of a mucus meniscus (blue). Left inset: Capillary adhesion between two flat, solid plates. Right inset: Capillary adhesion between a solid sphere and a flat, solid plate. **B** Hypothesised changes in wetting state with an increase in substrate roughness or pad-substrate gap width [97]. *d*_g_ gap width, *F*_⊥,cap_ capillary adhesion, *R* sphere radius, *R*_mer_, *R*_azi_ meridional and azimuthal radius of meniscus curvature, *β* filling angle, *γ* mucus surface tension, *ϕ* contact angle. B modified after [97]. Printed with permission

#### Attachment control

Several morphological elements in the limbs and digits of tree frogs are likely to contribute to an active control of attachment. The forelimbs are adapted towards an arboreal lifestyle. Specifically, kinematic and electromyographic analyses in *L. caerulea* and *Phyllobates bicolor* revealed that variations in the concerted action of the forelimb musculature allow for a power grip (i.e. clamping an object between flexed digits and palm), a precision grip (i.e. pinching an object between digit tips), and active positioning of the hands during climbing on narrow substrates [61]. A single layer of smooth muscle cells is present in the wall of each mucus gland [31, 37]. This muscle type accommodates large strains and might enhance the deformability of the glands, minimising unintentional mucus secretion during pad loading. A dermal nerve plexus probably innervates the glandular muscle cells and thus controls mucus squeeze-out [51]. Several authors [33, 62, 63] reported smooth muscle fibres in tree frogs’ toe pads, which, however, was not confirmed in later literature [31, 37].

The mucus ducts are surrounded by several layers of tightly interconnected cells [31], which support the ducts mechanically and presumably facilitate mucus squeeze-out. The dermal tissue between the terminal phalanx and the ventral epidermis is heavily vascularised (Fig. 1B 2; [44]), which might allow an active modification of pad curvature [64] and pad stiffness [47] by varying blood pressure.

#### Force transmission

The morphological basis of the transmission of attachment forces, generated at the pad-substrate interface, within the pad or to other body parts has not been studied extensively. An internal skeleton is the principle load bearing and transmitting structure in each limb (Fig. 1A 2,3). Many tree frog species have a cartilaginous intercalary element between the terminal and subterminal phalanx of each digit, which increases digit flexibility and facilitates axial rotations of the terminal phalanx (Fig. 1A 2,3; e.g. [6, 65, 66]). In each digit, two tendons support the skeleton in load transmission: the dorsal *Tendo Superficialis* extends the digit, and a ventral tendon connected to the musculus extensor brevis profundus adducts the terminal phalanx (Fig. 1A 2; [6, 66]).

Collagen fibres connect the terminal phalanx with the ventral basement membrane [44, 62, 65]. The low lateral connectivity of the collagenous structures (Fig. 1A 3) suggests a low stiffness of the pad in dorso-ventral compression and lateral extension [31]. The deformable lymph space [34] and the blood-vessel network in the connective tissue also point towards low stiffness and viscoelastic properties of the pad.

The lateral membranes of adjacent epidermal surface cells are interconnected basally, which mechanically strengthens the epidermis (Fig. 1C 2; [23, 37, 51]). Furthermore, tonofilament bundles—arranged parallel to the longitudinal axes of the superficial epidermal cells [52]—interconnect the cells through desmosomes [23, 31], split up towards the ventral surface, and terminate at the apical ends of the nanopillars (Fig. 1D 2; [23, 34, 38, 51]). The ordered arrangement of the tonofilaments vanishes, as they extend into the deeper epidermal layers [23]. We expect that tonofilaments, collagenous structures, and digital bones together facilitate the transmission of attachment forces from the pad-substrate interface to the rest of the body (e.g. for locomotion). The local expression of keratins forming the tonofilaments in the nanopillars [67] supports the relevance of the tonofilaments for force transmission. A thin layer of electron dense material covers the inner side of the plasma membrane of the apical cells (Fig. 1D 2; [23, 51]).

### Material properties

The high compliance of the toe pad in compression influences its attachment performance, for example by increasing the effective contact area on rough substrates. Compliance depends, among other properties, on the material-specific Young’s modulus *E*, on the Poisson’s ratio *ν*, and on geometry and spatial arrangement of load-bearing structures. Overall, the toe pads were reported to be very soft [65], with an effective elastic modulus *E*^∗^ = *E*/(1 − *ν*^2^) of the whole epidermis reported to be lower than that of most biological materials (e.g. [68]). Repeated indentation experiments showed no plastic deformation of the pad [22]. A small load-unload hysteresis in the force-displacement curve [22] and a decrease in the normal contact force during constant pad deformation [47] suggest viscoelasticity of the pad.

The effective elastic modulus of the toe pad varies by factors of up to 10^4^ between studies (Table 1). Such variations might be explained by the structure of the cytoskeleton [22], which makes *E*^∗^ strongly dependent on the location and direction of indentation, by the use of in vivo (e.g. [22]) versus *ex vivo* (e.g. [69]) samples, and by the use of different indenter shapes and contact mechanics models (e.g. Oliver-and-Pharr-theory in [22]; Johnson-Kendall-Roberts-/Hertz-model in other studies). Variations might also indicate a stiffness gradient [47] based on an increase of *E*^∗^ with indentation depth *d*_i_. The exact variation of *E*^∗^ with *d*_i_ is unknown.

**Table 1:** Experimental findings on the stiffness of tree frogs’ toe pads

Reference	Species	Effective elastic modulus *E*^∗^	*d*_i_	*r*_i_	Remarks
		[kPa]	[µm]	[µm]	
[21]	*L. caerulea*	33.5 ± 4.1	0.2	—	*Ex vivo*, AFM, Hertz theory
	*R. prominanus*	28.7 ± 10.5			
[22]	*L. caerulea*	14000	1.6	—	Pyramidal AFM tip
[47]	*L. caerulea*	4–25	200	—	Spherical MT, JKR-model
[69]	*L. caerulea*	54 ± 7	0.5	0.4	*Ex vivo*, Spherical AFM, Hertz theory
		40.7 ± 3.2		13.3	

*d*_i_ indentation depth, *r*_i_ indenter radius, AFM atomic force microscopy, JKR Johnson-Kendall-Roberts, MT microtribometry

### Mucus properties

The mucus forms a liquid bridge with a meniscus that fully surrounds the toe pad [46] and has a wedge thickness of 5–10 µm [21]. The meniscus height and curvature are unknown. The mucus viscosity *μ* in *L. caerulea* is about 1.43 mPas, measured with laser-tweezer micro-rheometry [25]. The mucus is often approximated as a Newtonian liquid (i.e. *μ* is strain-rate-independent), but non-Newtonian liquid properties are suggested by the presence of polysaccharides in filled mucus glands in *H. cinerea* [31]. The static contact angle *ϕ* of mucus microdroplets on hydrophilic and hydrophobic substrates is low (*ϕ*≪10°), which indicates an adhesive capillary function of the mucus independent of the wetting properties of the substrate [70].

## Functional demands on a toe pad

Morphology and operation of an attachment organ are codetermined by the functional demands on the respective organ [15]. In the tree frog, these demands arise, among others, from the environment, phylogeny, and lifestyle of the animals.

### Directional contact forces

Directional contact forces allow tree frogs to climb into the higher ecological layers of forests and other vegetation [71]. To stay attached to substrates with different inclination angles (e.g. overhanging leafs and vertical tree stems), tree frogs have to generate both strong adhesion and friction. The transmission of contact forces via skeletal elements suggests preferential directions of the contact force vector for whole limbs and single digits and thus anisotropic mechanisms of force generation. Gripping as a special case of force directionality is discussed elsewhere [39, 61, 72, 73].

### Substrates with diverse surface characteristics

Tree frogs encounter a variety of substrates such as plant leaves, tree bark, insect cuticle, and stones, with a wide range of random or structured roughness [68, 74, 75], surface energy (i.e. the energy required to form a unit area of free surface of a given material; [2]), stiffness [1], and wetting^2^ level. Natural substrates can be wetted via rain (e.g. in tropical habitats) and the mucus secretion on amphibian skin [72, 76, 77]. Environmental temperature, air humidity (e.g. [78]), and (mechanical or chemical) surface pollution may also affect the attachment performance of tree frogs. The ability of tree frogs to clean their pads by repeated stepping was discussed by Crawford et al. [71]. Generating contact forces that are high enough to keep the animals attached to natural substrates with different properties is arguably a primary demand on the toe pads.

### Static and dynamic attachment

Tree frogs use a combination of locomotory modes such as jumping, horizontal walking, and vertical climbing [17], for which reversible and repeatable attachment is crucial [1]. For dynamic conditions, attachment and detachment (and switching between the two states; [79]) should be fast and controlled [80], and contact forces need to be large enough to resist detachment from the substrate during sudden events such as the attack of a predator or the wind-induced shaking of a leaf [81]. Additionally, toe pads enable static attachment, as observed in resting frogs [13, 33] or during copulation [76].

### Transmission of contact forces

We expect toe pads to transmit the generated forces internally and to other body parts. Force transmission within a morphological unit, for example the epidermis, has been suggested to distribute mechanical stresses at the pad-substrate interface, hence reducing the risk of unwanted detachment [2] or of damaging the epidermis [15]. Force transmission between the epidermis and other body parts allows (directed) locomotion and requires a functional integration of the pads into the whole locomotory apparatus ([61, 66]; see also the functional morphology of force transmission), as observed in geckos [82].

## Basic theory of potential attachment mechanisms in a toe pad

Various mechanisms of force generation [24], as well as lubrication [25] and drainage of the secreted mucus [83] have been suggested to play a role in the attachment and detachment of tree frogs. Here, we introduce these mechanisms for the subsequent discussion of their possible contributions to attachment. For a list of the used symbols and for a discussion of suction as potential adhesion mechanism, we refer to Additional file 1.

### Force generation

#### Capillary forces

A liquid bridge in the gap between the toe pad and the substrate can be formed by the secretion of mucus, by capillary condensation of water vapour, or by external surface wetting (e.g. rain droplets). The meniscus of this bridge can cause capillary contact forces (Fig. 3A), arising from the surface tension *γ* of the liquid [84]. Capillary adhesion is attractive for a concave meniscus if seen from the gas phase (i.e. contact angle *ϕ*< 90°); for water, a circular, concave meniscus is present on a hydrophilic substrate up to a meniscus height *κ* = (*γ*/*gρ*)^−0.5^≈ 2.7 mm (*g* gravitational acceleration, *ρ* density).

A circular meniscus between two smooth, flat, rigid plates with equal contact angles (Fig. 3A, left inset) and with homogeneous surface energies is the first and most common model of capillary adhesion applied to tree frogs’ toe pads (e.g. [24]). According to this model, the capillary adhesion *F*_⊥,cap_ generated by a meniscus with azimuthal and meridional radii of curvature *R*_azi_ and *R*_mer_, respectively, is [2]:

1 $$\begin{array}{@{}rcl@{}} F_{\perp\text{,cap}} &=& 2 \pi R_{\text{azi}} \gamma \sin \phi + \pi R_{\text{azi}}^{2} \gamma \left(\frac{1}{R_{\text{mer}}} - \frac{1}{R_{\text{azi}}}\right)\\ R_{\text{mer}} &=& \frac{d_{\mathrm{g}}}{2 \cos \phi}. \end{array} $$

The first term represents the direct action of surface tension at the three-phase contact line (negligible at *R*_azi_≪*R*_mer_), and the second term the effect of the Laplace pressure across the meniscus surface. In reality, the contact angle *ϕ* can differ strongly from the ideal case assumed in the described models, as a result of phenomena such as contact-line pinning, surface energy variations due to substrate roughness, or the entrapment of air between a rough substrate and a fluid meniscus (Fig. 3B; [85]).

The capillary adhesion between a rigid sphere (radius *R*) and a flat plate may represent tree frog attachment more closely than the plate-plate contact. For equal contact angles and a filling angle *β* between the vertical and the three-phase contact line (Fig. 3A, right inset; [84]), Eq. 1 can be rewritten to model the sphere-plate contact:

2 $$\begin{array}{*{20}l} F_{\perp\text{,cap}} &= 2 \pi R \sin \beta \gamma \sin \left(\phi + \beta \right)\\&\quad + \pi R^{2} \sin^{2} \beta \gamma \left(\frac{1}{R_{\text{mer}}} - \frac{1}{R_{\text{azi}}}\right) \\ R_{\text{mer}} &= \frac{R \left(1 - \cos \beta \right)}{2 c} \\ R_{\text{azi}} &= R \sin \beta - R_{\text{mer}} \left[1 - \sin \left(\phi + \beta \right)\right] \\ c &= \frac{\cos \left(\phi + \beta\right) + \cos \phi}{2}. \end{array} $$

For *R*≪*R*_azi_≪*R*_mer_ and *β*,*ϕ*≈0, Eq. 2 simplifies to:

3 $$\begin{array}{@{}rcl@{}} F_{\perp\text{,cap}} &=& 4 \pi R \gamma. \end{array} $$

The capillary adhesion between two deformable objects (one of them, for example, being a deformable sphere, which may represent a soft, round toe pad more closely than a rigid, flat plate) is stronger than between two rigid objects, because of an increased contact area in the former case [86, 87]. For a discussion on the capillary adhesion of deformable objects and on capillary friction we refer to Additional file 1.

#### Hydrodynamic forces

**Fig. 4:**
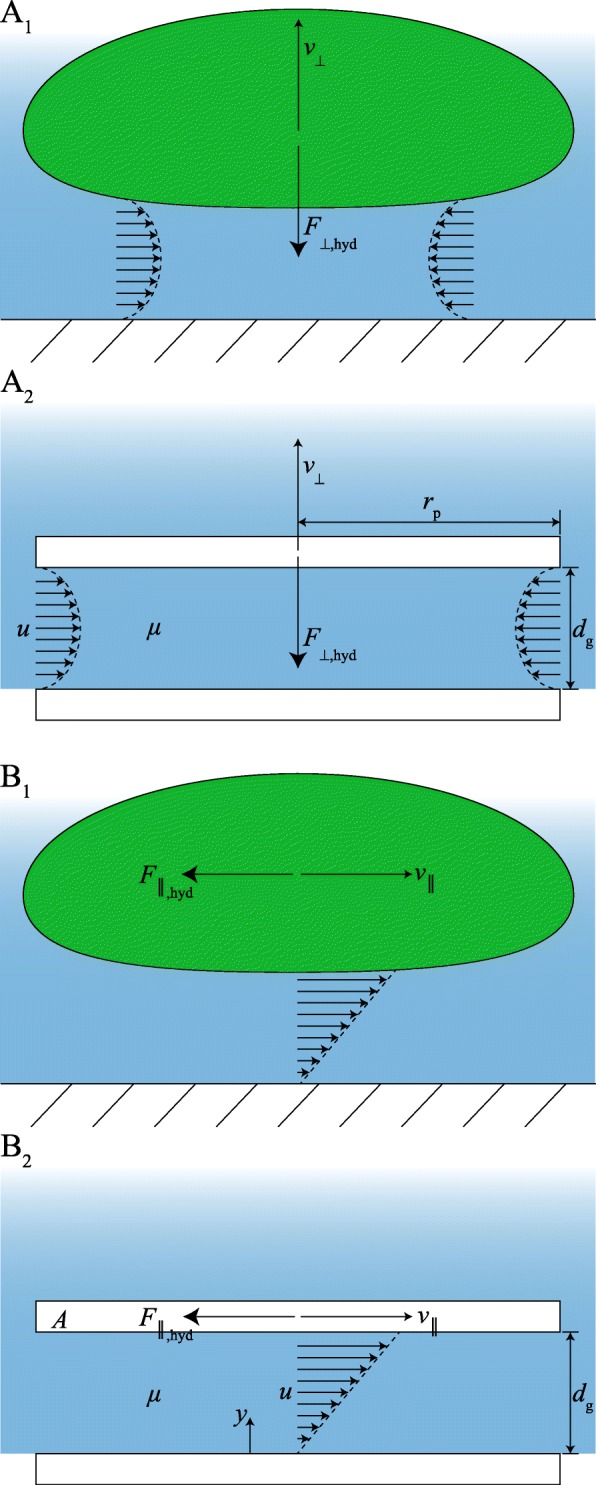
Hydrodynamic (**A**) adhesion and (**B**) friction (1) during the schematic interaction between a toe pad (green) and the substrate based on displacement-induced flow of mucus (blue) and (2) in a model of the contact of two flat and rigid (cylindrical) plates. *A* area, *d*_g_ gap width, *F*_⊥,hyd_ hydrodynamic adhesion, *F*_∥,hyd_ hydrodynamic friction, *r*_p_ plate radius, *u* flow speed, *v*_⊥_ detachment speed, *v*_∥_ sliding speed, *y* spatial coordinate normal to the substrate, *μ* viscosity

Mucus flow between toe pad and substrate during attachment and detachment generates hydrodynamic contact forces (Fig. 4). Hydrodynamic adhesion (also called Stefan or viscous adhesion) can be modelled assuming a flow between two flat, rigid plates with radius *r*_p_ fully immersed in a viscous liquid and initially separated by a distance *d*_g_ (Fig. 4A 2; [88]). During separation of the plates, liquid flows from the surroundings into the widening gap. Hydrodynamic adhesion *F*_⊥,hyd_ is the force required to overcome the viscous resistance against this flow [88, 89]:

4 $$\begin{array}{@{}rcl@{}} F_{\perp\text{,hyd}} &=& - \frac{\partial d_{\mathrm{g}}}{\partial t} \frac{3}{2} \pi \mu \frac{{r_{\mathrm{p}}}^{4}}{{d_{\mathrm{g}}}^{3}}. \end{array} $$

Whereas the attachment of two plates may represent the contact of a flattened pad with a substrate reasonably well, we expect that a sphere-plate contact describes the approach of a submerged, curved pad to the substrate better. Between a smooth sphere with radius *R* and a flat plate, *F*_⊥,hyd_ is [90]:

5 $$\begin{array}{@{}rcl@{}} F_{\perp,\text{hyd}} &=& - \frac{\partial d_{\mathrm{g}}}{\partial t} 6 \pi \mu \frac{R^{2}}{d_{\mathrm{g}}}. \end{array} $$

Hydrodynamic forces act oppositely to the direction of surface movement and can hence also be repulsive. Hydrodynamic repulsion during the approach of deformable objects is lower, and adhesion during separation is higher than for rigid objects [91].

Next to adhesion and repulsion, hydrodynamic effects can also cause hydrodynamic (viscous) friction. For the shear flow of liquid between a stationary plate (i.e. the substrate) and a plate sliding at a speed *v*_∥_ parallel to the stationary one (i.e. the toe pad; Fig. 4B 2), the hydrodynamic friction *F*_∥,hyd_ is [92]:

6 $$\begin{array}{@{}rcl@{}} F_{\parallel\text{,hyd}} &=& \mu A \frac{\partial u}{\partial y} \\ &=& \mu A \frac{v_{\parallel}}{d_{\mathrm{g}}}. \end{array} $$

Equation 6 is only valid for gaps large enough to allow free shear flow (with a linear velocity profile), and the concept of hydrodynamic friction should be applied with caution to tree frogs’ toe pads. It is likely that liquid is drained out of the pad-substrate gap during sliding, in which case Eq. 6 does not hold anymore, particularly with an increasing sliding distance. Alternatively, viscous-poroelastic effects have been proposed to contribute to tree frog attachment [93].

#### Van der Waals forces

**Fig. 5:**
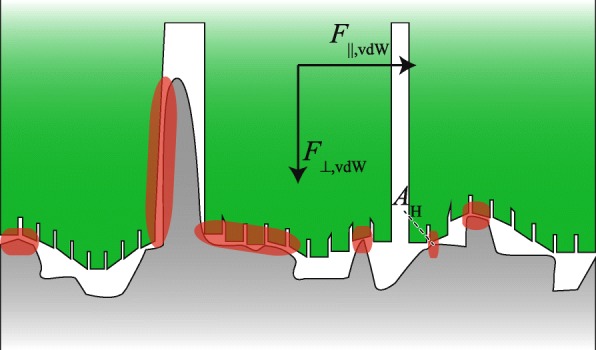
Schematic generation of van der Waals (vdW) forces (*F*_⊥,vdW_, *F*_∥,vdW_) between ventral toe pad epidermis (green) and substrate (grey) for a system-specific Hamaker constant *A*_H_. VdW interactions occur in regions of close pad-substrate contact (red)

Van der Waals (vdW) interactions between single atoms or molecules of a toe pad and the substrate may cause adhesive and frictional contact forces (Fig. 5). VdW forces are known to be dominant in the attachment of geckos (e.g. [82, 94]) and might also play a significant role in tree frogs [24, 25]. Between two flat plates with a contact area *A* separated by a distance *d*_g_, the macroscopic vdW force *F*_⊥,vdW_ is [95]:

7 $$\begin{array}{@{}rcl@{}} F_{\perp\text{,vdW}} &=& - A \: \frac{A_{\mathrm{H}}}{6 \: \pi}\frac{1}{{d_{\mathrm{g}}}^{3}}, \end{array} $$

where *A*_H_ is the system-specific Hamaker constant. *A*_H_ scales with the electron density of the interacting molecules and with temperature [96].

Friction arising from vdW interactions between two objects sliding along each other is termed dry (or Coulomb) friction. Dry friction *F*_∥,vdW_ is proportional to the normal load *F*_⊥,L_ (i.e. a body weight component *F*_⊥,g_ and, if applicable, adhesion *F*_⊥_) and the system-specific friction coefficient *μ*_∥_ [96]:

8 $$\begin{array}{@{}rcl@{}} F_{\parallel\text{,vdW}} &=& \mu_{\parallel} F_{\perp\text{,L}} \\ &=& \mu_{\parallel} \left(F_{\perp\text{,g}} + F_{\perp} \right). \end{array} $$

#### Mechanical interlocking

**Fig. 6:**
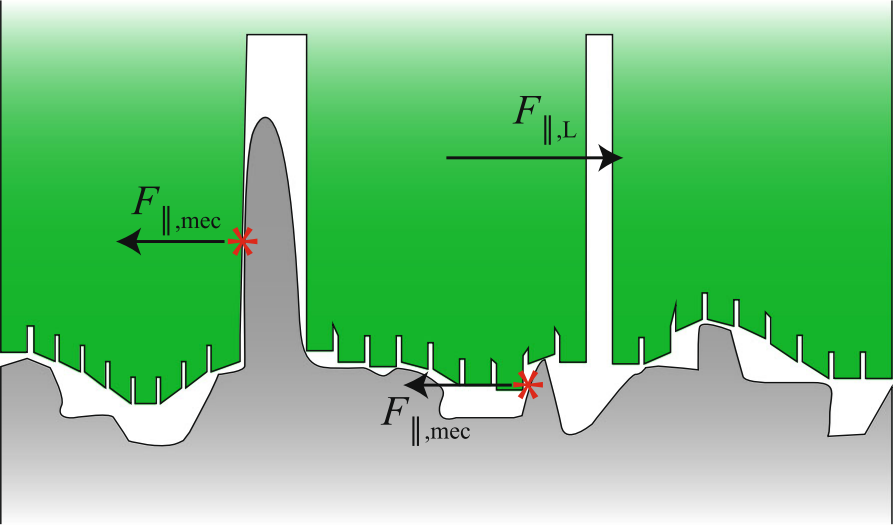
Schematic mechanical interlocking between a superficial cell (left star) or a nanopillar (right star) on the ventral toe pad epidermis (green) and asperities of a rough substrate (grey) at a shear load *F*_∥,L_

Mechanical interlocking is the mutual intermeshing of (parts of) an attachment organ and substrate asperities [3]. In tree frogs, interlocking between the epidermal cells and the asperities of a rough substrate has been proposed to contribute to attachment (Fig. 6; [24, 97]). Arguably, the attachment force generated by mechanical interlocking is proportional to the number of individual contact points.

### Liquid management

The mucus between toe pad and substrate not only introduces hydrodynamic or capillary forces, it may also lubricate the pad during sliding and hinder closure of the pad-substrate gap requiring drainage of surplus mucus. Here, we introduce the theories of lubrication and drainage with respect to their potential appearance in tree frog attachment.

#### Lubrication

**Fig. 7:**
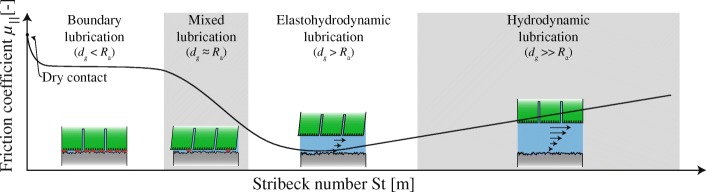
Stribeck diagram showing the proposed spectrum of lubrication modes and the resulting friction coefficients *μ*_∥_ in tree frogs’ toe pads as a function of the Stribeck number St

Lubrication of an object sliding over a substrate with velocity *v*_∥_ changes the generated friction dramatically compared to dry friction (Fig. 7). The regime of lubrication of the pad-substrate-system depends on its Stribeck number St=(*μ*_∥_*v*_∥_)/(*F*_⊥,L_ *A*^−1^) [98]. At low St, dry pad-substrate contacts and dry friction dominate (boundary lubrication; Equation 8). At higher St (e.g. lower normal load *F*_⊥,L_ per unit area), dry contacts between substrate asperities and the sliding toe pad become less frequent, and the load of the pad is carried both by dry contacts and enclosed volumes of mucus (mixed lubrication). At even higher St, the mucus carries most of the load, and the pad and substrate influence each other by deformation of substrate asperities through the mucus (elastohydrodynamic lubrication). At large St, loads are transmitted only via the mucus layer (hydrodynamic lubrication), and hydrodynamic friction occurs (Eq. 6).

#### Drainage

**Fig. 8:**
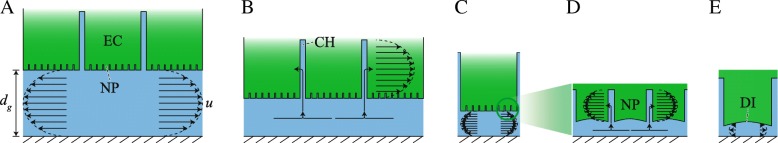
Hypothesised regimes of mucus drainage in a tree frog’s toe pad. **A** Far field regime. **B** Intermediate field regime. **C** Near field regime. **D** Drainage through the nanochannel network and **E** the nanopillar-substrate gap. DI dimple, EC epidermal cell, NP nanopillar. *d*_g_ gap width, *u* flow speed. Modified after [99]. Printed with permission

In artificial adhesives [99, 100], and possibly also in tree frogs, a channel network, which is separated by liquid from a substrate, leads to several drainage regimes depending on the gap width *d*_g_. In the nomenclature of these regimes, we follow Gupta & Fréchette [99]. For *d*_g_≫*d*_0_ (=*w*_c_ (*h*_c_/(*w*_c_+*d*_c_))^1/3^≈ 1 µm in tree frogs; [99, 100]), radial squeeze-out of liquid through the gap (far field regime; Fig. 8A) was confirmed experimentally in artificial surfaces covered with cylindrical pillars [99]. For *d*_g_≈*d*_0_, liquid flows increasingly through the channels, which become the main source of hydrodynamic friction, down to a distance *d*_1_ (intermediate field regime; Fig. 8B). For *d*_g_≪*d*_0_, the viscous resistance against liquid flow between single pillars and substrate dominates (near field regime; Fig. 8C). Drainage in tree frogs through the nanopillar channels and single nanopillar-substrate gaps may be assessed analogously to the drainage through microscopic artificial surface structures (Fig. 8D, E; [83]). In torrent frogs, the epidermal channel system is elongated along the proximal-distal pad axis [8, 11]. This elongation may ease the drainage of water flowing around the toe pads, hence enabling the strong attachment of these animals on overflowed substrates [8–12].

## Attachment performance of tree frogs

The adhesive and frictional performance of tree frogs have been studied for whole animals and single limbs or toe pads. Adhesion and friction of whole frogs have been typically measured using a platform that rotates around a horizontal axis (Tables 2 and 3, top; Additional file 1: Figure SI.3), originally designed by Emerson & Diehl [24] and refined by Hanna & Barnes [6]. Simple trigonometry allows a calculation of adhesion and friction based on the measured inclination angles at which the animals slide on (*α*_∥_) and fall off (*α*_⊥_) the platform (see Additional file 1). For single limb/pad-measurements, various force transducers (Tables 2 and 3, bottom) have been used. Effects of substrate properties on attachment forces have been also measured and behavioural traits related to attachment have been observed.

**Table 2:** Measured adhesion performance of whole tree frogs (top) and of single limbs/toe pads (bottom; SP unless stated otherwise) on smooth dry substrates

Reference	Species	Adhesion *F*_⊥_	Tenacity *σ*_⊥_	Remarks
		[mN]	[mN mm ^−2^]	
[6]	*O. septentrionalis*	75.5	1.2	PMMA
[24]	*H. cinerea*	39.24	1.4	Teflon
[40]	various hylids	2.0–372.0	0.4–1.3	PMMA
[41]	various hylids	4.3–180.2	0.4–0.7	PMMA
[42]	various hylids	0.5–200.0	0.30–1.08	PMMA
[71]	*L. caerulea*	255.3 ± 73.7	—	Glass
[6]	*O. septentrionalis*	5.9 ± 2.1–14.9 ± 3.6	—	SL
[10]	*R. pardalis*	—	1.5	PE
[39]	*R. dennysi*	1.7–11.3	1.1–2.3	Varying detachment kinematics
[65]	*H. arborea*	127.53	—	SL, Frontlimb, Metal
[71]	*L. caerulea*	—	1.08 ± 0.24	Glass
[97]	*L. caerulea*	—	1.74 ± 1.90	Resin
		—	1.43 ± 0.60	PDMS
[101]	*L. caerulea*	—	0.04–1.12	Varying load angle
[113]	*L. caerulea*	13.9–34.0	—	SL

PE polyethylene, PMMA polymethyl-methacrylate (acrylic glass), SL single limb measurement, SP single pad measurement

**Table 3:** Measured friction performance of whole tree frogs (top) and of single limbs/toe pads (bottom; SP unless stated otherwise). For explanation of abbreviations see Table 2

Reference	Species	Friction *F*_∥_	Shear stress *σ*_∥_	*F*_⊥,L_	*v*_∥_	Remarks
		[mN]	[mN mm ^−2^]	[mN]	[µm s^–1^]	
[40]	various hylids	5.5–585.7	—	—	—	PMMA
[71]	*L. caerulea*	285.4 ± 94.5	—	—	—	Glass
[6]	*O. septentrionalis*	24.9 ± 6.6–55.4 ± 6.3	—	—	—	SL
		3.0–130.3	—	2.5	10–2500	
[10]	*R. pardalis*	—	1.5	—	—	Glass
[25]	*L. caerulea*	—	1.08–2.01	0.1	500	Glass
[39]	*R. dennysi*	25.1–51.2	9.6	2	600	Varying detachment kinematics
[46]	*H. versicolor*	357.1	—	—	—	Pulling experiment, Frontlimbs
[53]	*P. megacephalus*	17.52	—	3	300	Glass
[80]	*T. resinifictrix*	110–1270	—	—	—	Jumping kinematics, Wood; SL
[97]	*L. caerulea*	—	7.8 ± 12.9	2	1000	Resin
		—	5.9 ± 2.6			PDMS
[113]	*L. caerulea*	1.6–10.4	—	—	—	SL
[112]	*P. megacephalus*	14.5–122.7	—	—	—	Repeated sliding

*F*_⊥,L_ normal load, *v*_∥_ sliding speed

Here, we address findings on the attachment performance of tree frogs with respect to the questions stated in the introduction: Which mechanisms do contribute to tree frog attachment and how does the pad morphology support these mechanisms? We attempt to answer these questions by finding the best possible interpretation of the previous findings with regard to the pad properties, functional demands and, particularly, to the above described mechanisms, for example by comparison of measured contact forces with model predictions. Potential key questions and approaches for future developments in the field will be described in the final section.

### Adhesion

#### Measured adhesion performance

##### Whole animals

The adhesion measured for whole tree frogs ranges between 0.5 and 372 mN (Table 2, top) and scales above squared with snout-vent-length *ℓ*_SV_(*F*_⊥_∝*ℓ*_SV_^2.19^;[42]). Body mass *m* scales roughly volumetrically (i.e. isometrically) with , whereas the ventral pad area *A* scales approximately quadratically with . The resulting negative scaling of contact area per body mass with body size [41, 42] leads to a decline in adhesive performance with body size [24, 40–43]. Adhesion scales as *F*_⊥_∝*A*^1−1.19^ [24, 40, 43] and at a higher rate with *ℓ*_SV_ than *A* [41], which is favourable compared to the situation of isometric scaling. For a discussion of potential adaptations to the problem of isometric scaling, see Smith et al. [42].

Despite a variation of the measured adhesion by a factor of 10^4^, the tenacity (i.e. adhesive force per unit area) measured for whole tree frogs on smooth substrates varies relatively little, between 0.3 and 1.4 mN mm^–2^ (Table 2, top). In these calculations, however, contact area was assumed to equal the total ventral area of all toe pads (e.g. [6, 41]), whereas during the actual rotating platform experiments the frogs tend to change the number and size of pad contacts. Therefore, the maximum tenacity is presumably underestimated, and accordingly the goodness of fit of the interspecific tenacity scaling by Smith et al. [42] with body size (*r*=0.78, *p*=0.04) and epidermal cell size is low (*r*=0.81–0.92, *p*=0.003–0.02, min. 1 animal for *A*_p_, min. 10 animals for *F*_⊥_). Furthermore, a significant intraspecific correlation of *σ*_⊥_ with *ℓ*_SV_ is not found in all tree frog species [41].

##### Single limbs/pads

Tenacities measured in single pads agree with whole animal tenacities (Table 2, bottom). Endlein et al. [39] reported an effect of the detachment kinematics on tenacity: proximal pulling on the pads before detachment led to higher tenacities compared to detachment in a dabbing movement. Similarly, Barnes et al. [101] measured in *L. caerulea* a negative scaling of the tenacity with the pull off angle *θ*_L_ between substrate and pulling force from 1.12 mN mm^–2^ at 53° to 0.04 mN mm^–2^ at 170°, pointing towards peeling of the toe pads.

##### Local indentations

In adult *L. caerulea*, Barnes et al. [47] measured normal pull-off forces (i.e. adhesion) of 585–609 µN using a spherical indenter with radius *r*_i_ = 1.5 mm at indentation depths *d*_i_ ≈50−350 µm. Assuming a surface area *A*=2*πd*_i_*r*_i_ for the spherical cap of the indenter in contact with the pad, we computed tenacities of 0.17–1.29 mN mm^–2^, which overlaps with the values reported above. Similarly, Kappl et al. [69] measured adhesion of 5 nN in dead *L. caerulea* using a spherical AFM-indenter (*r*_i_= 13.3 µm) at *d*_i_≈ 250−300 nm for submerged pads (i.e. no capillary force generation), from which we calculated tenacities of 0.12–0.24 mN mm^–2^.

#### Adhesion mechanisms

##### Capillary adhesion

Tree frog adhesion has been attributed primarily to wet adhesion considering that: (i) Mucus fills the pad-substrate gap and forms a capillary meniscus [24]. (ii) Nachtigall [102] measured for two glass plates separated by distilled water a capillary tenacity of 7 mN mm^–2^, which is in the same order of magnitude as tenacities measured for tree frogs. (iii) Tree frog adhesion scales linearly with *A*, as predicted by capillary adhesion based on Laplace pressure (Eq. 1; [24]), assuming a size-invariant meridional meniscus curvature. (iv) On rough substrates, adding liquid improves the adhesive performance, proposedly by sustaining the meniscus.

**Fig. 9:**
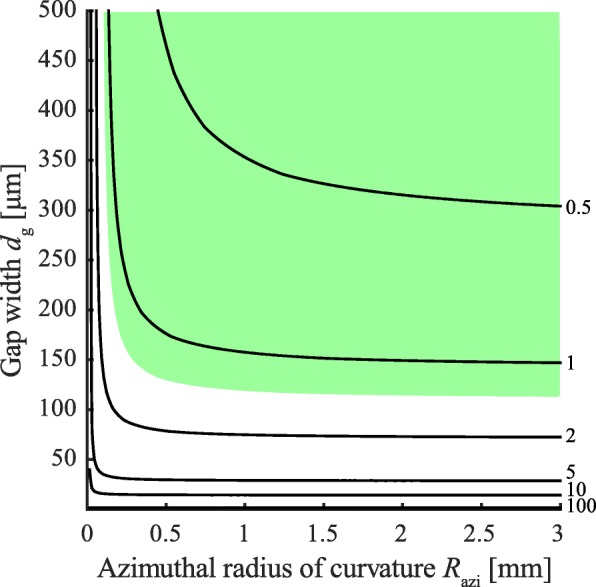
Tenacity contours [mN mm ^−2^] computed for capillary adhesion at varying gap widths (i.e. twice the meridional radius of meniscus curvature) and azimuthal radii of meniscus curvature (≈0.5 *d*_p_), respectively, according to Eq. 1. We assumed *ϕ*= 0° and *γ*= 71.97 mN m^–1^. The green patch shows the combinations of *R*_azi_ and *R*_mer_ that lead to tenacities in the range of measured values [47]

To theoretically investigate the role of capillary adhesion, we calculated the capillary tenacity for various combinations of meniscus curvatures (i.e. meniscus height and pad diameter). Since the adhesion between a sphere and a plate (Eq. 3) does not show the area-scaling measured in tree frogs [24, 40, 43], we modelled the pad-substrate interaction as plate-plate contact (Eq. 1). As shown in Fig. 9, a meridional radius of meniscus curvature *R*_mer_ similar in size to the micro- to nanoscopic height *d*_g_ of the mucus film (e.g. 5 µm estimated in [40]) would lead to capillary adhesion that is several orders of magnitude higher than the tenacities measured in tree frogs. In reality, the meniscus covers also the side of the pad [21] and therefore *R*_mer_≫*d*_g_/2 (compare Fig. 3A, left inset). Thus, Eq. 1 might well describe tree frog adhesion under the assumption of a realistic radius of meniscus curvature that is much larger than the narrow pad-substrate gap width. Based on Fig. 9, we predict *R*_mer_ ≈ 150 µm. As discussed by Drechsler & Federle [103], we would expect a minimisation of the radii of meniscus curvature (i.e. just enough mucus to fill the pad-substrate gap as found in artificial structured adhesives [70]) in pads adapted towards capillary adhesion.

Figure 9 further shows that, depending on pad size, both meniscus curvatures have to considered in computing the capillary adhesion of tree frogs’ toe pads. To our knowledge, models of the capillary adhesion of tree frogs, such as the ones discussed above or in previous works (e.g. [6, 7, 24, 104]), do not take into account variations in the contact angle (and hence of meniscus curvature) related to wetting phenomena such as contact-line pinning or substrate roughness [85].

The linear scaling of adhesion with contact area [41] is not only explained by capillary adhesion. For example, such scaling might also originate from suction, mechanical interlocking, or vdW forces, assuming a uniform load-distribution over the contact area. In contrast to capillary effects, the latter two mechanisms might directly explain the friction of tree frogs’ toe pads.

With respect to morphology, the micro- to nanoscopic channel system has been suggested to support capillary adhesion by quickly spreading the mucus over the pad surface for a rapid formation of the liquid bridge [9, 83]. In addition, the channels may facilitate the capillary condensation of water vapour into the pad-substrate gap, reducing the need to actively secrete mucus. However, the distance at which a capillary bridge forms between substrate and an artificial adhesive covered with a channel network is reduced, presumably because liquid is redistributed from the liquid bridge into the channels [70]. Accordingly, channels could also counteract quick generation of capillary adhesion, particularly if there is only little liquid present in the pad-substrate gap.

##### Hydrodynamic adhesion

Hydrodynamic models predict an above-area scaling of adhesion with *ℓ*_SV_, which disagrees with the area-scaling measured in tree frogs. Therefore, viscosity-based forces are not believed to play an important role in tree frog adhesion [6, 24]. Due to its inherent rate-dependency, hydrodynamic adhesion might prevent rapid detachment [41], which would inhibit, for example, jumping. Other adhesive mechanisms such as capillary adhesion or vdW forces do not show such an inherent rate-dependency [105]. Furthermore, hydrodynamic adhesion requires continuous pad movements, rendering this mechanism ineffective against continuous forces such as gravity. For a deformable pad, gap closure (and hence the formation of potential dry contacts or of low gap widths for strong hydrodynamic adhesion) presumably is even slower compared to a rigid one [91]. In other words, hydrodynamic adhesion seems more of a hindrance for the animal (i.e. attachment and detachment are retarded and adaptations towards control of hydrodynamic forces may be needed), rather than a primary mechanism of adhesion.

The empirical and modelling evidence of hydrodynamic adhesion in the soft and patterned toe pads of tree frogs is limited. Current analytical models assume the contact of rigid objects. With decreasing stiffness, fluid-structure interactions increasingly affect hydrodynamic adhesion [91], and contact forces resulting from viscoelastic substrate deformations can even exceed the hydrodynamic forces [106]. Moreover, current models assume smooth surfaces. Modified hydrodynamic boundary conditions are needed to model flow over structured surfaces [107]. Further work is required to examine if current analytical models of hydrodynamic adhesion can represent tree frog attachment accurately.

##### Van der Waals forces

**Fig. 10:**
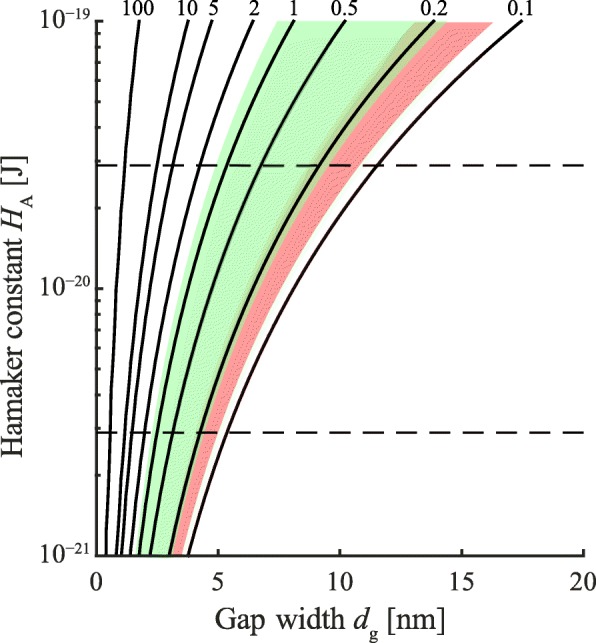
Tenacity contours [mN mm ^−2^] computed for van der Waals (vdW) interactions at various Hamaker constants and gap widths according to Equation 7. We assumed that 10% of the contact area contributes to vdW force generation. Dashed lines: Theoretical minimum Hamaker constant for water separating two similar materials at 26 °C (bottom) and a 10-fold higher Hamaker constant (top). Coloured patches show the combinations of *A*_H_ and *d*_g_ leading to tenacities in the range of measured values ([47], green; [69], red)

Previously, large gap widths and a decrease of *A*_H_ as a result of the liquid present in the pad-substrate gap were stated to prevent any significant contribution of vdW forces in tree frog attachment [24]. To examine the possibility of vdW forces in tree frogs, we calculated the vdW-tenacity using Eq. 7 for various combinations of pad-substrate gap width *d*_g_ and Hamaker constant *A*_H_ for a range of values expected for tree frogs’ toe pads (Fig. 10; max. *A*_H_ ≈ 10^−19^ J in dry conditions [96]; min. *A*_H_≥0.7 *k*_b_*T*=2.9·10^−21^ J for water between two similar materials at temperature T = 26° and Boltzmann constant *k*_b_ = 1.4·10^−23^ J K^–1^). Even in a conservative prediction using *A*_H_ = 2.9·10^−21^ J and an effective contact area of 10% of *A*, this model yields vdW tenacities equal to or higher than 1 mN mm^–2^ (see Additional file 1) at *d*_g_≤2.5 nm on a smooth substrate. For a 10-fold higher Hamaker constant, which is in the range of values reported for two dissimilar organic objects interacting across water [96, 105], vdW forces are equal to or higher than the adhesion measured in tree frogs at *d*_g_≤ 6.7 nm. Using interference reflection microscopy, Federle et al. [25] measured *d*_g_≤ 5 nm for more than 40% (and *d*_g_≤ 10 nm for more than 55%) of the analysed epidermal cells. This sensitivity analysis suggests that tree frogs are potentially able to conform close enough to the substrate to generate significant vdW forces despite liquid in the pad-substrate gap and a reduced Hamaker constant.

Morphological observations also support the action of vdW forces in toe pads. The accumulation of electron dense material in the outermost layer of the nanopillars (Fig. 1D 2) could increase vdW interactions [105], analogously to the effects of varying thicknesses of the substrate backing material on the vdW forces reported for geckos [94].

Summarizing, the contribution of vdW forces to adhesion cannot be excluded in tree frogs (although more experimental evaluation is needed). Quantifications of the Hamaker constant, the pad-substrate gap width, and the attachment performance on substrates with different surface energies or with chemically different backing layers (as performed for geckos in [94]) are required for a detailed assessment of the contribution of vdW forces to tree frog attachment.

##### Drainage

Most mechanisms described in this review predict an increase in adhesion (and friction) with decreasing pad-substrate gap width. Liquid in the pad-substrate gap impedes a close conformation of the pad, and adaptations towards liquid drainage might be at play. The different drainage regimes could help explain the function of the micro- to nanoscopic channel network in between the ventral epidermal cells and nanopillars. These channels might effectively enlarge the gap width [83], and reduce hydrodynamic repulsion (Eqs. 4 and 5). Thus, drainage would alleviate a reduction of the gap width and reduce the duration of contact formation (and separation). The grip to the substrate would be closer and faster, as demonstrated for artificial surface structures [53, 70, 99, 100]. As described above, the flow through the channel network is dominated by viscous effects [99, 100].

### Friction

#### Measured friction performance

Table 3 summarizes the friction *F*_∥_ and shear stress *σ*_∥_ measured for whole tree frogs and single limbs/toe pads. Static friction exceeds adhesion in terms of force [9] and stress [39, 97]. The static friction coefficient *μ*_∥_ of a pad ranges between 0.77 and 1.98 in various species tested on PMMA [9, 40]. For single toe pads, Chen et al. [53] measured that friction during sliding along the longitudinal pad axis exceeds the friction of lateral sliding by ca. 20%. Kappl et al. [69] reported a contradictory trend of 29–71% higher friction coefficients for lateral sliding of single epidermal cells. Friction scales with *ℓ*_SV_ just below cubed (*F*_∥_∝*ℓ*_SV_^2.76−2.78^; [9, 40]), indicating an approximately linear scaling with body mass. Federle et al. [25] measured a static shear stress of 1.12 mN mm^–2^ two minutes after the end of sliding, which was explained by boundary lubrication (i.e. dry friction).

Friction dynamics are hardly studied in tree frogs. Single pad friction scales positively with sliding velocity in *Osteopilus septentrionalis* [6]. In *L. caerulea*, a (median) dynamic shear stress of 2.1 mN mm^–2^ was reported [25]. Dynamic peak friction values of up to 1270 mN, equivalent to 14.4 times the body weight, were reported for single pads of *Trachycephalus resinifictrix* [80]. We expect that the high frictional performance reported in recent studies [39, 53, 80, 97] can be explained by a scaling of friction with normal load and shear velocity.

#### Friction mechanisms

##### Mechanical interlocking

Interlocking of epidermal cells or nanopillars with substrate asperities might contribute to friction (and adhesion) of tree frogs [46]. Interlocking might explain the enhanced attachment forces measured on substrates covered with artificial pillars similar in size to the epidermal cells (and channels; [97]). An enhanced contact area for dry or hydrodynamic friction might be an alternative explanation of this observation.

Overall, interlocking, as described for stiffer attachment organs such as claws [108, 109], is debatable for the delicate epidermal cells of tree frogs. The use of substrates with a well-defined topography (e.g. structured or random roughness as in [97]) is crucial in future investigations.

##### Lubrication: from dry to hydrodynamic friction

The measured mass- (and therefore load-) scaling of friction [40], the observation of static friction, and nanoscopic pad-substrate gap widths [25] indicate the presence of dry friction. However, the measurement of a lower static than dynamic friction [25] conflicts with dry friction and suggests the action of additional friction mechanisms.

For example, the presence of mucus and the positive scaling of friction with sliding velocity point towards hydrodynamic friction (or possibly rubber friction; [9, 95]). Physiological adaptations towards enhanced hydrodynamic friction could target the mucus viscosity, velocity gradients, and contact area: the wetted contact area, which is considerably larger than the projected area, and high velocity gradients because of the nanoscopic pad-substrate distances may enhance hydrodynamic friction, despite a low mucus viscosity.

Overall, we expect that tree frogs experience the whole lubrication spectrum from dry to hydrodynamic friction, with boundary lubrication as preferred regime of lubrication, because it provides static friction, which is load-dependent and hence controllable.

Lubrication might also explain the large amount of glands secreting mucus into the pad-substrate gap. Compared to geckos [82], the surface of tree frogs’ toe pads is keratinised only little and is accordingly very soft. While this facilitates the uptake of water and oxygen through the skin [110] and enhances the substrate conformability, we also expect the soft pads to be more susceptible to abrasive wear [95]. A thin layer of mucus (i.e. a few layers of mucus molecules) might act as lubricant to avoid excessive damage of the pad epidermis while maintaining a sufficiently high pad friction.

##### Friction anisotropy

Lubrication could also cause the anisotropic friction of polygonal surface structures observed in toe pads [53], with a higher friction in the longitudinal direction than in the lateral direction, and in artificial surfaces [12], where the friction of a regular pattern of regular hexagonal pillars is 60°-symmetric: friction anisotropy might arise from direction-dependent liquid flow in the channel network because of anisotropic channel alignment [53], or from the anisotropic geometry and bending stiffness of the surface structures [12]. Furthermore, we propose that the anisotropic channel alignment could lead to anisotropic sliding velocities and a direction-dependent transition to another lubrication regime (and friction coefficient). In tree frogs, high friction along the proximal-distal pad axis seems most important, as suggested by yawing motions of the toe pads in jumping frogs before landing [80], which agrees with anisotropic friction predicted by the theories of anisotropic flow.

### Effects of variations in substrate properties on attachment

#### Measured effects

##### Roughness

**Fig. 11:**
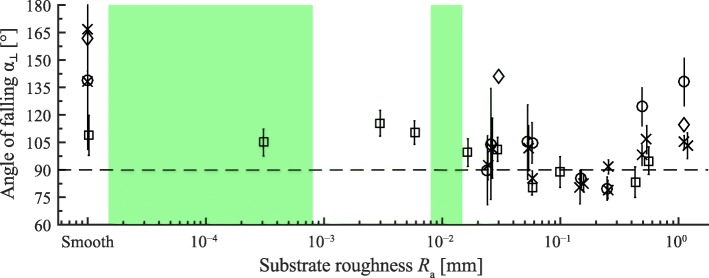
Variation of the falling angle *α*_⊥_ with substrate roughness *R*_a_ on an inclined, dry substrate (circles from Fig. 5 in [43], *Hyla microcephala*; crosses from Fig. 8A in [9], *Colostethus trinitatis*; diamonds from Fig. 3B in [10], *Rhacophorus pardalis*; squares from Fig. 1B in [97], *Litoria caerulea*). Green areas denote the diameter range of nanopillars (left) and epidermal cells (right) reported in the main text. Dashed line: Falling angles below 90° show the lack of adhesive abilities. Most roughness values mentioned in the references are approximations and do not originate from measurements

In Fig. 11, we provide an overview of tree frog adhesion as a function of the (arithmetic) average roughness *R*_a_ of the substrate. For *R*_a_< 6 µm, the tenacity was reported to increase compared to a smooth surface [97]. Crawford et al. [97] also measured a higher tenacity on a substrate with structured roughness (i.e. 3 µm high and 2 µm wide pillars with variable spacing) than on a smooth substrate. With increasing pillar spacing, the tenacity returns to the values measured for smooth substrates at a spacing ≥ 10 µm. Tree frogs tend to adhere worse to rough substrates [43], for example wood or coarse sandpaper, which was also observed for smooth substrates contaminated with glass beads with a diameter of 50 µm [71]. At *R*_a_≥0.5 mm, adhesion increases again.

Effects of *R*_a_ on friction have been hardly studied. Compared to various other substrates, in ten species the highest friction coefficients were found on wood [9]. Similar to adhesion, higher shear stresses were measured on substrates with *R*_a_ = 3 − 6 µm than on smoother substrates [97]. For microscopic glass beads (diameter < 3 µm), Crawford et al. [97] observed interlocking of the beads in the intercellular epidermal channels.

##### Wetting

Tree frogs cannot attach to a fully wetted, smooth substrate [13, 24, 65, 111]. Endlein et al. [10] described a reduction of *σ*_⊥_ from ca. 1.5 mN mm^–2^ to ca. 0.1 mN mm^–2^ when wetting the pad with 10 µL of water, which is equivalent to a 2.3 mm thick liquid film assuming an average pad surface of 4.3 mm^2^. On rough substrates, on the other hand, light wetting (i.e. rates of 0.8–1.9 mL s^–1^ of water flowing over the test substrate or spraying water on the substrate) increases adhesion compared to dry conditions [9, 10, 43, 97]. Crawford et al. [97] reported the ability of *L. caerulea* to fill gaps—created by 50−75 µm large glass beads contaminating the substrate—with mucus. For even larger beads, air bubbles formed in the pad-substrate gap.

‘Full’ wetting weakens friction on smooth and rough substrates [10]. In repeated friction measurements on individual toe pads (10 consecutive steps), Zhang et al. [112] measured an increase of friction with step number (from 14.5 mN to 122.7 mN), presumably because of a reduction of the liquid volume between pad and substrate. Similar to adhesion, friction on a rough substrate (58.5 µm) increases by light wetting [97].

##### Surface energy/tension

The only study investigating the influence of substrate surface energy on adhesion reported a difference of the mean falling angle by 9° in frogs sitting on glass versus teflon [24]. A reduction in surface tension of the intervening liquid by wetting a substrate with water mixed with a detergent led to a complete loss of friction [46].

#### Functional interpretation of measured effects

##### Capillary adhesion

The reduced adhesion of tree frogs on dry, rough substrates has been attributed to the formation of bubbles within substrate cavities (Fig. 3B 3), reducing the contact area available for capillary adhesion [43]. However, meniscus cavitation around glass beads in the pad-substrate gap occurs only for bead diameters > 50 µm [97], leaving the reduced adhesion at lower roughnesses unexplained by reduced capillary forces. In addition, capillary adhesion is unlikely to explain the increased adhesion on rough substrates for *R*_a_ ≤ 6 µm [97], unless the low contact angle is further reduced because of roughness. These observations point towards the action of other adhesion mechanisms that are affected by microscopic roughness levels.

The loss of adhesion on a fully wetted substrate may result from a complete destruction of the meniscus and of capillary force generation [24]. Alternatively, strong wetting could widen the pad-substrate gap, weakening all potentially involved mechanisms.

The enhanced adhesion on rough substrates by light wetting has been explained by ‘filling’ of substrate cavities and by the preservation of the liquid bridge (i.e. capillary adhesion; [43]). However, stronger capillary adhesion may also lead to a reduction of the pad-substrate gap width or an enlargement of the area of dry contact, hence indirectly enhancing other mechanisms of force generation, which are likely to be weakened at the tested roughnes levels of ca. 30–60 µm [10, 97].

##### ‘Dry’ adhesion mechanisms

The enhanced adhesion on substrates with asperities ≲6µm may be explained by mechanical interlocking [97]. One could also attribute this finding to an enhanced contact area and vdW forces. The seemingly continuous decrease of *α*_⊥_ shown in Fig. 11 for *R*_a_= 2–60 µm suggests a continuous reduction of the effective ‘dry’ contact area and vdW forces with increasing roughness (in contrast to an expected drop of capillary adhesion because of meniscus cavitation at a critical roughness). Also, the weakened adhesion on rough (i.e. a reduced effective contact area) as well as wetted smooth substrates (i.e. a wider pad-substrate gap and a reduced Hamaker constant) and the scaling of tenacity with the number of beads contaminating the pad-substrate gap (i.e. a reduced effective contact area) reported in [71] are consistent with vdW forces. The enhanced adhesion on very rough substrates (*R*_a_≥0.2 mm) might be created by the whole toe pad interlocking with macroscopic substrate projections.

##### Friction mechanisms

Similar to adhesion, the increase of friction on microrough compared to smooth substrates may originate from mechanical interlocking [97]. Alternatively, an increase in effective contact area and vdW forces could explain this finding. The negative correlation between friction and the mucus volume present in the pad-substrate gap suggested by the findings of Endlein et al. [10] and Zhang et al. [112] may originate from the inverse scaling of hydrodynamic friction with mucus film thickness. Alternatively, more (and stronger) dry contacts could explain this observation.

### Behavioural adaptations and attachment control

Studying the use of a pad (and of other body parts) during locomotion provides insight into the dynamic mechanisms of force generation and attachment control in tree frogs. For example, the digits in the forelimbs perform a proximal pulling movement during attachment in normal walking [6, 62]. Such movements increase the attachment force [39], possibly because of an increased contact area, enhanced mucus spread, a more uniform load distribution, or pad cleaning [71].

On an increasingly tilted substrate, *L. caerulea* begins to splay the initially adducted limbs until all appendages are maximally extended [10, 113]. Limb splaying reduces the angle *θ*_L_ between substrate and pulling force, which is aligned approximately with the limb, and thus increases the force at which the pad peels off (see Additional file 1); sliding of the limbs and an increase of *θ*_L_ because of gravity result in the need to continuously reposition the fore- and hindlimbs, which explains the ‘dance-like’ movements of tree frogs on overhanging substrates [10].

During attachment, tree frogs rely not only on their pads but also on portions of belly and thighs to create contact forces [6]. At a slope of 90°, belly and thigh form 73% of the total contact area [10]. However, on an overhanging substrate the contact area is largely formed by the toe pads [10]. Torrent frogs show a different behaviour, with an increasing contribution of belly and thighs to the contact area with an increase of the substrate inclination from 90° to 180° [10], highlighting the importance of considering animal behaviour while studying the attachment performance of whole animals.

The toe pads detach from the substrate during normal walking in a peeling motion from posterior to anterior [6]. Detachment is probably controlled by the tendons and muscles [6], and peeling occurs passively when the pad is pulled from the substrate. On a vertical substrate, tree frogs that are rotated around the sagittal body axis realign their bodies towards the vertical axis, presumably to avoid passive peeling [6, 40].

## Conclusions and perspectives

Studies on morphology, material, and attachment forces of the toe pads of tree frogs have contributed considerably to the understanding of the attachment of these animals. We offer a systematic review of these studies, facilitating an in-depth discussion of the mechanisms involved in the generation, transmission, and control of attachment forces in the toe pads. Research which integrates contributions from experimental and computational biomechanics, physics, biochemistry, morphology, ecology, phylogenetics, and biomimetics is required to further deepen our understanding of tree frog attachment. Simultaneously, the discussion on tree frog attachment should be extended beyond isolated theories and pad features. An overarching model is needed, which integrates the functional demands on the toe pads, the pad morphology, and the various mechanisms contributing to time-dependent adhesion and friction. Here, the formulation and testing of a systematic series of hypotheses may help to identify the mechanisms to be considered. Moreover, we emphasise the role of friction in tree frog attachment. Recent works (e.g. [25, 39, 69, 113]) have highlighted the importance of friction in tree frog attachment and future studies on toe pad friction may advance the understanding of the functioning of tree frog toe pads. Due to the diversity of the involved phenomena, achieving an understanding of tree frog attachment arguably is even more complicated than for the attachment in geckos. Below, we outline possible contributions from various disciplines which may improve our understanding of tree frog attachment.

We argue that the pad-substrate contact area has been overestimated in previous research [80, 97], pointing towards an underestimation of the adhesive and frictional performance of tree frogs. In addition, tenacity and shear stress of so-called subarticular/digital tubercles—more proximal regions of digital epidermis covered with surface structures similar to those on the pads—were measured to be on average 3.2–8.8 times higher compared to the pads [39]. The difference in attachment performance between the toe pads and tubercles is not yet explained.

It is likely that tree frogs rely on several attachment mechanisms, and that the relative importance of these mechanisms varies with the circumstances [24], because tree frogs have to interact with a wide diversity of natural surfaces, requiring static and dynamic, adhesive and frictional, reversible, and repeatable force generation. In addition, tree frog species vary greatly in size (with ranges of snout-vent-length and body mass covering up to two orders of magnitude; [42]). Next to variations of substrate properties, the attachment apparatus of tree frogs has to deal with this scaling.

A liquid bridge in the pad-substrate gap most likely enables capillary adhesion. Current analytical plate-plate models overpredict the generated capillary adhesion by several orders of magnitude, sphere-plate models do not predict an area-scaling of adhesion, and experimental findings (e.g. pad-substrate gap widths ≤5 nm and increased adhesion on microrough compared to smooth substrates) indicate the involvement of other adhesion mechanisms. To directly evaluate the applicability of current models of capillary forces in tree frog attachment, we propose the simultaneous measurement of the capillary pressure within the meniscus and of meniscus parameters such as height (i.e. curvature), diameter, and contact angle.

Hydrodynamic adhesion is hard to predict by analytical models. This mechanism seems more of a hindrance for the animal (e.g. slower attachment and detachment), rather than a primary mechanism of adhesion. We suggest a combination of the visualisation of the mucus flow under a real pad (e.g. via micro-particle-image-velocimetry), the development of a computational fluid dynamics model including fluid-solid interactions, and measurements on artificial hierarchically structured surfaces to further investigate the mucus flow dynamics and the resulting hydrodynamic forces in tree frogs.

A sensitivity analysis shows that even in a conservative computation van der Waals (vdW) forces could contribute to tree frog adhesion. Measurement of the attachment performance on substrates with similar surface energies but different subsurface energies, as done for geckos [94], may help to conclude on the possibility of vdW forces contributing to tree frog attachment.

Mechanical interlocking and suction cannot be ruled out as additional adhesion mechanisms. Variation of the environmental pressure should directly affect the hypothetical suction of tree frog toe pads. In line with previous experiments [24, 62], we suggest single pad force measurements in a pressure chamber to test whether suction is present.

A simultaneous measurement of the three-dimensional contact force exerted by a single pad over a whole step cycle, contact area, and pad deformation is still missing. Such an observation is crucial for enhancing the understanding of the fundamentals of tree frog attachment. In such an experiment, control—or at least reporting—of experimental parameters such as normal load, sliding speed, contact area, or gap width is crucial, as presented in recent studies (e.g. [39, 114]). This also includes a characterisation of the used test substrates and of environmental conditions (e.g. temperature and air humidity). Also, the amount of mucus in the pad-substrate gap should get controlled, for example by bringing the pad in contact with a glass slide under defined conditions (as shown for insects [79, 115]). Comprehensive control and reporting of the experimental conditions may facilitate future meta-analyses on the attachment performance of tree frogs.

With respect to the effects of the variation of substrate properties on tree frog attachment, only roughness has been tested extensively. In geckos, the effects of variations in surface and subsurface energy as well as stiffness of the substrate have been analysed experimentally [94, 116]. Analogously, we suggest systematic variations of substrate properties (e.g. roughness and surface energy), properties of the wetting liquid (e.g. surface tension and viscosity), and simultaneous measurements of adhesion, friction, and contact area of single pads in combination with variations of preload, sliding speed, and detachment speed, to determine the full range of the attachment performance of tree frogs. In particular, a parametric friction study with control of normal load, sliding speed, and gap width would help to establish the dependence of friction on the Stribeck number (i.e. the Stribeck curve). Such a study could give a better understanding of the involved tribological mechanisms (e.g. the role of the mucus in friction) and might allow for conclusions on liquid and material properties [98] of the pad by comparison to Stribeck curves of known systems.

In contrast to geckos, tree frogs secrete mucus, which presumably fulfils various functions: (i) epidermal water and oxygen uptake, (ii) capillary and hydrodynamic force generation, (iii) avoidance of pad stiffening and a reduced conformability, and (iv) lubrication and hence reduction of abrasive wear. Studying the mucus properties is central to elucidating tree frog attachment.

Considering the effects of variations of roughness and wetting on tree frog attachment, we propose an interplay of vdW and capillary forces depending of substrate roughness: On low-roughness substrates (*R*_a_≤10–20 µm), vdW forces could be dominant and any liquid between pad and substrate would weaken this adhesion component. On rougher substrates, a liquid filled pad-substrate gap may support capillary adhesion, which then could (partially) compensate for the expected reduction of vdW forces. Based on this hypothetical interplay, we predict a trade-off in force generation based on capillarity and vdW interactions: Liquid filling the pad-substrate gap enhances capillary forces, but too much liquid reduces the Hamaker constant and vdW interactions.

Morphology and material composition of the toe pad suggest a high conformability to the substrate (i.e. reduced pad-substrate gap width and enhanced effective contact area) and consequently the importance of dry contacts: The hierarchical surface pattern on the ventral pad surface presumably reduces the effective bending stiffness of the surface and increases the wetted area of the pad. The intercellular channel network, the convex pad curvature, and macroscopic grooves on the ventral epidermis may facilitate viscosity-dominated drainage of interstitial liquids and gap-closure in the central part of the pad, and inertia-dominated drainage in the grooves and in the peripheral contact region, unaffected by the presence of epidermal surface structures (similar to, for example, car tires; [83]). Furthermore, the pads are very soft, enabling the conformation to a rough substrate. Barnes et al. [47] reported a negative spatial stiffness gradient from the pad surface towards deeper tissues. Analysing the shape of this gradient may help to understand the conformability and distribution of mechanical stresses within the pad epidermis. Considering the load transmitting elements such as tonofilaments, we expect a higher tensile stiffness than the compressive stiffnesses reported in Table 1. The effects of such a variable elastic modulus or of potential non-Hookean material properties (e.g. strain stiffening) on force generation are unknown.

The trajectories of the epidermal tonofilaments presumably facilitate force transmission through the epidermis, but how are forces transmitted from there to the phalanx and to other body parts? We suggest a detailed morphological analysis (employing histology, immunohistochemistry, and micro-computer-tomography) of tree frogs’ toe pads, with focus on force-transmitting structures, such as cytoskeletal elements, connective tissue, and muscular tissue. Such an analysis may show (i) where exactly contact forces are generated, (ii) which types of loading are dominant in the pads, and (iii) whether shear stiffening, as observed in geckos [28, 117], is common in both dry and wet adhesives. We expect that force transmission within the epidermis limits local stress concentrations, enhancing adhesion [2].

Little is known about active components and attachment control in the toe pads. Can tree frogs actively modify the geometry of the epidermal channels to control, for example, hydrodynamic force generation? Do the pads facilitate energy recovery during take offs from compliant substrates [118]? The muscular complex in the limbs of tree frogs suggests that tree frogs can control alignment of the pads and parameters such as normal and shear loading forces and speeds, thus regulating attachment force generation.

Next to capillary friction and suction (see Additional file 1), there may be other attachment mechanisms that have not yet been identified or have been only hypothesised. For example, Bijma et al. [80] explained the high friction of toe pads wrapped around curved substrates by capstan friction (i.e. the increased holding force of a rope wrapped around a winch). Friction of a soft pad could also partially arise from pad deformations due to surface tension at the three-phase contact line. During pad sliding, a movement of the contact line might induce dynamic pad deformations leading to energy dissipation in the potentially viscoelastic pad [119]. Furthermore, viscoelasticity of the soft pads may affect friction independently of the presence of a meniscus [120]: In so-called rubber friction, substrate roughnes causes dynamic deformations of the toe pad. Thus, during sliding, energy is continuously dissipated in the material, which can be seen as friction resisting sliding [9, 95].

The toe pads of tree frogs can serve as a model system for the design of biomimetic adhesives, inspiring novel versatile attachment solutions. A deeper understanding of the attachment mechanisms and functional advantages of the hierarchically structured ventral pad surface (i.e. epidermal cells and nanopillars) could further advance the design of biomimetic adhesives. In this light, we propose a comparative examination of intraspecific differences in habitat, attachment performance, and morphology of the whole pad as well as the epidermal surface structures, as done in geckos [121, 122]. One could expect, for example, fore-hindlimb differences in morphology and attachment performance because of different functions (hindlimbs serve in jumping [123]; forelimbs serve as shock absorbers [124]). Furthermore, adhesion was reported to correlate with cell size [42]. Although not fully substantiated, the examination of such a correlation may lead to new insights in the role of the different attachment mechanisms in tree frogs. Are cell geometry, size of the intercellular channels, or the stiffness of a cell (or a combination of these features) causal factors affecting attachment? What is the optimal geometry of the polygonal surface structures? The hierarchical surface structures on the ventral toe pad epidermis, the scaling of attachment performance with cell size, and the potential presence of vdW forces in tree frog attachment may also hint towards the working of contact splitting (i.e. the increase of attachment force resulting from subdivision of the contact area), which has been discussed for various biological and technical attachment systems [2, 60, 125–127]. Further work is required to elucidate the importance of contact splitting in tree frogs. We expect that the design of biomimetic adhesives will benefit significantly from addressing these issues.

## Additional file

Additional file 1 **Symbols and abbreviations**. List of symbols, List of abbreviations. **Morphology and material properties of a toe pad**. Geometrical model of the surface of the ventral toe pad epidermis, Fibre-matrix analogy of a toe pad, Scaling of tenacity with snout-vent-length. **Attachment performance of tree frogs**. The rotating platform experiment, Capillary adhesion of deformable objects, Capillary friction, Suction, Kendall peeling model, Johnson-Kendall-Roberts model. (DOCX 125 kb)

## Footnotes

1 ‘Holding’ and ‘attaching’ synonymously describe combinations of adhesion and friction, whereas ‘adhering’ exclusively refers to adhesion.

2 ‘Wetting’: the presence of liquid on a substrate.
