# Supplementary material for: Tree frog attachment: mechanisms, challenges, and perspectives
Source: Front Zool. 2018 Aug 23;15:32. doi: 10.1186/s12983-018-0273-x (PMC6107968; doi:10.1186/s12983-018-0273-x)
Supplement: Supplementary file 1 — Symbols and abbreviations. List of symbols, List of abbreviations. Morphology and material properties of a toe pad. Geometrical model of the surface of the ventral toe pad epidermis, Fibre-matrix analogy of a toe pad, Scaling of tenacity with snout-vent-length. Attachment performance of tree frogs. The rotating platform experiment, Capillary adhesion of deformable objects, Capillary friction, Suction, Kendall peeling model, Johnson-Kendall-Roberts model. (DOCX 125 kb) [file 12983_2018_273_MOESM1_ESM.docx]

**Tree frog attachment: Mechanisms, challenges, and perspectives**

Supplementary information

Julian KA Langowski^1*^, Dimitra Dodou^2^, Marleen Kamperman^3^, Johan L van Leeuwen^1^

^1^ Experimental Zoology Group, Department of Animal Sciences, Wageningen University & Research,
PO Box 338, 6700AH Wageningen, The Netherlands.

^2^ Department of BioMechanical Engineering, Faculty of Mechanical, Maritime and Materials Engineering,
Delft University of Technology, 2628CD Delft, The Netherlands.

^3^ Physical Chemistry and Soft Matter,Wageningen University & Research,
PO Box 0838, 6700EK Wageningen, The Netherlands.

^*^ Correspondence (E-mail: julian.langowski@wur.nl).

**Contents**

[SI Supplementary information 2](#_Toc500345842)

[1 Symbols and abbreviations 2](#_Toc500345843)

[a List of symbols 2](#_Toc500345844)

[b List of abbreviations 4](#_Toc500345845)

[2 Morphological and material properties of a toe pad 5](#_Toc500345846)

[a Geometrical model of the surface of the ventral toe pad epidermis 5](#_Toc500345847)

[b Fibre-matrix analogy of a toe pad 6](#_Toc500345848)

[c Scaling of tenacity with snout-vent-length 7](#_Toc500345849)

[3 Attachment performance of tree frogs 8](#_Toc500345850)

[a The rotating platform experiment 8](#_Toc500345851)

[b Capillary adhesion of deformable objects 8](#_Toc500345852)

[c Capillary friction 9](#_Toc500345853)

[d Suction 9](#_Toc500345854)

[e Kendall peeling model 10](#_Toc500345855)

[f Johnson-Kendall-Roberts model 11](#_Toc500345856)

[References 12](#_Toc500345857)

[Figures 14](#_Toc500345858)

# SI Supplementary information

## 1 Symbols and abbreviations

### a List of symbols

**Table SI.1:** List of Roman (top) and Greek (bottom) symbols in alphabetical order.

| **Symbol** | **SI Unit** | **Description** |
| --- | --- | --- |
| *A* | m^2^ | Ventral surface area of all toe pads / Contact area |
| *a*_c_ | m | Edge length of the hexagonal, apical surface of an epidermal cell |
| *A*_c_ | m^2^ | Apical surface area of an epidermal cell |
| *A*_c,c_ | m^2^ | Projected area of an epidermal cell and the surrounding channel |
| *A*_c,w_ | m^2^ | Wetted area of an epidermal cell and the surrounding channel |
| *A*_H_ | J = kg m^2^ s^−2^ | Hamaker constant |
| *A*_n,eff._ | m^2^ | Apical surface area of a nanopillar uncovered by a dimple |
| *A*_eff_ | m^2^ | Apical surface area of all nanopillars uncovered by a dimple, i.e. potential contact area for vdW interactions |
| *A*_p_ | m^2^ | Ventral surface area of a single toe pad (i.e. apparent contact area) |
| *b* | m | Tape width |
| *d*_c_ | m | Approximate diameter of the hexagonal, apical surface an epidermal cell |
| *d*_g_ | m | Width of gap between substrate and an object (e.g. toe pad or synthetic adhesive) |
| *d*_i_ | m | Indentation depth |
| *d*_n_ | m | Approximate diameter of a nanopillar |
| *d*_p_ | m | Diameter of the contact area of a single toe pad |
| *d*_0_, *d*_1_ | m | Critical gap widths in drainage flow |
| *E*_(f,m,c)_ | Pa = kg m^−1^ s^−2^ | Young’s modulus [of the fibre (_f_) and matrix (_m_) fraction of a composite material (_c_)] |
| *E*_s_ | Pa = kg m^−1^ s^−2^ | Effective elastic modulus of a system of two contacting objects |
| *E*^*^ | Pa = kg m^−1^ s^−2^ | Effective elastic modulus |
| *F*_L_ | N = kg m s^−2^ | Load |
| *F*_m_ | N = kg m s^−2^ | Body weight |
| *F*_⊥_ | N = kg m s^−2^ | (Maximum) adhesion, generated by capillary effects (_cap_), hydrodynamic effects (_hyd_), vdW interactions (_vdW_), mechanical interlocking (_mec_), or suction (_suc_) |
| *F*_⊥_*_,_*_g_ | N = kg m s^−2^ | Body weight component normal to surface |
| *F*_⊥_*_,_*_L_ | N = kg m s^−2^ | Normal load |
| *F_\|\|_* | N = kg m s^−2^ | (Maximum) friction, generated by capillary effects (_cap_), hydrodynamic effects (_hyd_), vdW interactions (_vdW_), mechanical interlocking (_mec_), or suction (_suc_) |
| *F_\|\|,_*_g_ | N = kg m s^−2^ | Body weight component parallel to surface |
| *F_\|\|,_*_L_ | N = kg m s^−2^ | Shear load |
| *g* | m s^−2^ | Gravitational acceleration |
| *h* | m | Film thickness |
| *h*_c_ | m | Height of a freestanding hexagonal cell |
| *h*_m_ | m | Height of artificial surface structure |
| *h*_n_ | m | Height of a nanopillar |
| *k*_b_ | J K^−1^ = kg m^2^ s^−2^ | Boltzmann constant |
| *k*_1_*,k*_2_ | var. | Constants used in curve fits |
| *ℓ*_SV_ | m | Snout-vent-length |
| *m* | kg | Body mass |
| *n*_c_ | - | Number of cells per toe pad |
| *n*_n,c_ | - | Number of nanopillars per cell |
| *n_n,_*_c_ | - | Number of nanopillars per toe pad |
| *p* | - | Probability value |
| *P*_in_ | Pa = kg m^−1^ s^−2^ | Suction pressure |
| *P*_env_ | Pa = kg m^−1^ s^−2^ | Environmental pressure |
| *r* | - | Coefficient of determination |
| *r*_i_ | m | Indenter tip radius |
| r_n_ | m | Dimple radius |
| *R* | m | Radius of curvature of a toe pad or sphere |
| *R*_a_ | m | Arithmetic average roughness |
| *R*_azi_ | m | Azimuthal radius of meniscus curvature |
| *R_mer_* | m | Meridional radius of meniscus curvature |
| *r*_p_ | m | Radius of cylindrical plate |
| *R*_s_ | m | Radius of curvature of a system of two contacting objects |
| St | - | Stribeck number |
| *t* | s | Time |
| *T* | K | Temperature |
| *u* | m s^−1^ | Flow speed |
| *v*_⊥_ | m s^−1^ | Normal detachment speed |
| *v*_\|\|_ | m s^−1^ | Sliding speed |
| *V*_f_ | - | Volume fraction of the fibres of a composite material |
| *w*_c_ | m | Width of a channel between two epidermal cells |
| *w*_n_ | m | Width of a channel between two nanopillars |
| *W*_⊥_ | J m^−2^ = kg s^−2^ | Work of adhesion |
| *α*_c_ | ° | Internal angle of a hexagonal epidermal cell |
| *α*_⊥_ | ° | Falling angle in rotation table experiments |
| *α*_\|\|_ | ° | Sliding angle in rotation table experiments |
| *β* | ° | Meniscus filling angle |
| *∆c* | m | Peeling distance |
| *∆P* | Pa = kg m^−1^ s^−2^ | Suction pressure difference |
| *∆r* | m | Distance between dimple perimeter and midpoint of an edge of a nanopillar |
| *∆γ* | N m^−1^ = kg s^−2^ | Fracture energy |
| *γ* | N m^−1^ = kg s^−2^ | Surface tension |
| *κ* | m | Maximum meniscus height for capillary condensation |
| *η* | - | Drainage efficiency factor |
| *θ_L_* | - | Angle between load and surface |
| *µ* | Pa s = kg m^−1^ s^−1^ | Dynamic viscosity |
| *µ_\|\|_* | - | Friction coefficient |
| *ν* | - | Poisson’s ratio |
| *ρ* | kg m^−3^ | Fluid density |
| *ρ*_c_ | m^−2^ | Density of epidermal cells per toe pad area |
| *ρ*_ch_ | m^−2^ | Length of channels between epidermal cells per toe pad area |
| *σ*_⊥_ | Pa = kg m^−1^ s^−2^ | Tenacity |
| *σ_\|\|_* | Pa = kg m^−1^ s^−2^ | Shear stress |
| *σ*_⊥,max_ | Pa = kg m^−1^ s^−2^ | Maximal tenacity |
| *φ*_(1,2,l,t)_ | ° | Contact angle between gas-solid (_1_) and gas-liquid interface (_2_) at leading (_l_) and trailing edge (_t_), respectively |

### b List of abbreviations

**Table SI.2:** List of abbreviations in alphabetical order.

| **Abbreviation** | **Description** |
| --- | --- |
| AFM | Atomic force microscopy |
| CI | Confidence interval |
| JKR | Johnson-Kendall-Roberts |
| MT | Microtribometry |
| PDMS | Polydimethylsiloxane |
| PE | Polyethylene |
| PMMA | Polymethyl-methacrylate |
| PVS | Polyvinylsiloxane |
| SE | Standard error |
| SEM | Scanning electron microscopy |
| SL | Single limb measurement |
| SP | Single pad measurement |
| TEM | Transmission electron microscopy |
| vdW | van der Waals |

## 2 Morphological and material properties of a toe pad

### a Geometrical model of the surface of the ventral toe pad epidermis

The symbols *b*, *c*, *d*, *e*, *l*, *β*_c_, and *r*_n_ are not listed in the table of symbols for the sake of clarity. An approximately circular toe pad with a diameter *d*_p_ has a ventral surface area *A*_p_ of:

| (SI.1) | $A_{\text{p}}=\left(\frac{d_{\text{p}}}{2}\right)^{2}$ |
| --- | --- |

Assuming an equilateral hexagonal outline, the projected apical surface area *A*_c_ of a single cell is (Fig. SI.1A):

| (SI.2a) | $A_{\text{c}}=2 b c+2 b a_{\text{c}}$ |
| --- | --- |

| (SI.2b) | $\alpha_{\text{c}}=2\arccos\left(\frac{c}{a_{\text{c}}}\right)$ |
| --- | --- |

| (SI.2c) | $b=a_{\text{c}}\sin\left(\frac{\alpha_{\text{c}}}{2}\right)$ |
| --- | --- |

| (SI.2d) | $c=a_{\text{c}}\cos\left(\frac{\alpha_{\text{c}}}{2}\right)$ |
| --- | --- |

The projected area covered by a cell and the surrounding channel *A*_c,c_ is:

| (SI.3a) | $A_{\text{c,c}}=2\left(b+d\right)\left(c+l-e\right)+2\left(b+d\right)\left(a_{\text{c}}+2 e\right)$ |
| --- | --- |
| (SI.3b) | $\beta_{\text{c}}=\arctan\left(\frac{\frac{a_{\text{c}}}{2}}{b}\right)$ |
| (SI.3c) | $d=\frac{w_{\text{c}}}{2}$ |
| (SI.3d) | $e=d \tan(\beta_{\text{c}})$ |
| (SI.3e) | $l=\frac{d}{\sin(\alpha_{\text{c}})}$ |

This gives ca. *n*_c_ cells per toe pad:

| (SI.4) | $n_{\text{c}}\approx\frac{A_{\text{p}}}{A_{\text{c,c}}}$ |
| --- | --- |

The wetted area *A*_c,w_, neglecting the nanopillars, of a single cell is:

| (SI.5) | $A_{\text{c,w}}=A_{\text{c,c}}+6 a_{\text{c}} h_{\text{c}}$ |
| --- | --- |

where *h*_c_ is the height of the free-standing cell. Equations SI.2a, SI.3a and SI.5 can be used analogously to calculate the different areas of a single nanopillar (assuming an equilateral hexagonal outline; Fig. SI.1B), which gives *n*_n,c_ nanopillars per cell and in *n*_n,p_ nanopillars per pad:

| (SI.6) | $n_{\text{n,c}}=\frac{A_{\text{c}}}{A_{\text{n,c}}}$ |
| --- | --- |
| (SI.7) | $n_{\text{n,p}}=n_{\text{c}} n_{\text{n,c}}$ |

We exclude the dimple on the apical nanopillar surfaces from the effective apical contact area of the whole toe pad in close contact to the substrate. Assuming a circular dimple on a nanopillar with a regular, hexagonal outline with a minimal distance ∆*r* between dimple perimeter and nanopillar edge centroid, the effective apical area is:

| (SI.8a) | $A_{\text{n,eff.}}=A_{\text{n}}-\pi r_{n}^{2}$ |
| --- | --- |
| (SI.8b) | $r_{n}=\frac{a_{\text{n}}\sqrt{3}}{2}-\Delta r$ |

The total ventral pad area effective for vdW force generation is:

| (SI.9) | $A_{\text{eff.}}=A_{\text{n,eff.}} n_{\text{n,p}}$ |
| --- | --- |

### b Fibre-matrix analogy of a toe pad

Scholz et al.


[1] proposed an analogy of the tree frog’s toe pad epithelium with a fibre-reinforced material. Assuming the layer of nanopillars to be a simple, unidirectional fibre-matrix-composite, the effective elastic modulus of the composite *E*_c_ is


[2]:

| (SI.10) | $E_{\text{c}}=E_{\text{f}}V_{\text{f}}+E_{\text{m}}(1-V_{\text{f}})$ |
| --- | --- |

The indices mark the elastic modulus *E* and the volume fraction *V* of the fibres (_f_) and the matrix (_m_). With this relation, we can compute the expected volume fraction of fibres for a given composite’s effective elastic modulus. We assume a composite stiffness of 14 MPa, as found for the keratinised layer


[1], and further a matrix stiffness of 20 kPa, as found for the whole pad (e.g.


[3]), which presumably is dominated by the underlying matrix (Table 1). With *E*^*^= 4 GPa for keratinous tonofilaments (e.g.


[4]), Equation SI.10 results in a fibre volume fraction of 0*.*2%. As shown in Fig. 1D_2_, the apical ends of the epithelial cells are densely packed with tonofilaments and 0.2% underpredicts the fibre content. The hypothesised asymmetric stiffness of the tonofilaments with a higher stiffness in tension than in compression most likely explains this deviation. In compression, the fibre stiffness *E*_f_ arguably will be arguably lower, for which Equation SI.10 predicts a higher fibre ratio *V*_f_ with *E*_f_.

### c Scaling of tenacity with snout-vent-length

Previously, the relation between tenacity *σ*_⊥_ and snout-vent-length *ℓ*_SV_ has been modelled linearly (e.g.


[5]). A linear model is not able to predict local optima or asymptotic limit values in the *σ*_⊥_-*ℓ*_SV_-relationship, thus possibly neglecting physical limitations (e.g. a maximum possible tenacity for a given attachment mechanism). As shown in Fig. SI.2, linear scaling of *σ*_⊥_ with snout-vent-length *ℓ*_SV_ results in *σ*_⊥_ = k_1_ + k_2_ *ℓ*_SV_ [where *k*_1_ = 0.45 ± 0.09 mN mm^−2^, *k*_2_ = 0.006 ± 0.001 mN mm^−3^, mean ± 95% confidence-interval (CI), Standard error (SE) = 0.23 mN mm^−2^] using total least squares regression. We quantified CI with a Monte Carlo approach (1000 iterations). One could argue that a relation converging to a limit value, for example an exponential growth decay model (), represents the tenacity scaling of tree frogs more closely than a linear one, which predicts unrealistically large adhesive tenacities in frogs larger than 10 cm. In the exponential growth decay model, *k*_1_ and *k*_2_ are fitting constants and *σ*_⊥_*_,_*_max_ is the maximum tenacity. By fitting this function to the maximum tenacities measured in tree frogs, we can compute a maximum adhesive tenacity *σ*_⊥_*_,_*_max_ = 0*.*88 mN mm^−2^ ± 0*.*07 mN mm^−2^ (standard error SE = 0*.*21 mN mm^−2^; Fig. SI.2).

## 3 Attachment performance of tree frogs

### a The rotating platform experiment

For a frog sliding/falling on/from a platform that rotates around a horizontal axis, adhesion *F*_⊥_, friction *F_||_*, and friction coefficient *µ_||_* calculate as follows from the angles of falling (*α*_⊥_) and slipping (*α_||_*; Fig. SI.3):

| (SI.11a) | $F_{\vert\vert}=m g \sin(\alpha_{\vert\vert})$ | $0< \alpha_{\vert\vert}<90$ |
| --- | --- | --- |
| (SI.11b) | $F_{\perp}=m g \cos(180-\alpha_{\perp})=-m g cos(\alpha_{\perp})$ | $90 < \alpha_{\perp}<180$ |
| (SI.11c) | $\mu_{\vert\vert}=\frac{F_{\vert\vert}}{m g \cos(\alpha_{\vert\vert})+F_{\perp}}=\frac{\sin(\alpha_{_{\vert\vert}})}{\cos(\alpha_{_{\vert\vert}})-\cos(\alpha_{\perp})}$ |  |

Here, *m* is the body mass and *g* is the gravitational acceleration.

### b Capillary adhesion of deformable objects

Butt et al.


[6] extended Equation 1 to model the capillary adhesion between a deformable, smooth sphere with radius *R* and a plate with contact angles *φ*_1_ (sphere-liquid) and *φ*_2_ (plate-liquid; Fig. SI.4), which they proposed to represent the capillary adhesion of a soft, curved toe pad more closely than a plate-plate contact (Fig. SI.4). A deformable sphere experiences stronger adhesion than a rigid one of the same dimensions, because of the larger contact area of the former. Assuming *R*≫*R*_azi_ ≫*R*_mer_, a large indentation depth compared to *R*_mer_, very hydrophilic materials (*φ*_1_ = *φ*_2_ ≈ 0), an effective elastic modulus of the system (i.e. pad-substrate) *E*_s_^*^ =*E*_1_^*^ + *E*_2_^*^, and an effective radius of the system *R*_s_ = *R*_1_ *R*_2_ / (*R*_1_ + *R*_2_), capillary adhesion can be calculated as


[6]:

| (SI.12) |  |
| --- | --- |

In the deduction of this model, indentation depths being much larger than the meniscus height are assumed, which may lead to unrealistic results in the case of tree frog attachment: the expected meniscus height (see main text and Fig. 9) is in the order of indentation depths reported for real animals (e.g.


[3]) and the meniscus shape might be affected by the indentation.

### c Capillary friction

Next to adhesion, capillary effects can also generate friction. Assuming a shear load acting on a pad with width *b*, the substrate-liquid contact angle shifts asymmetrically at leading (*φ*_l_) and trailing edge (*φ*_t_). The resulting capillary friction *F_||,_*_cap_ is


[7]:

| (SI.13) | $F_{\vert\vert\text{,cap}}=2 b \gamma\left(\cos\varphi_{\text{t}}-\cos\varphi_{\text{l}}\right)=4 b \gamma$ |
| --- | --- |

Equation SI.13 predicts a maximum shear stress of around 0.14 mN mm^−2^, which is below the stresses measured for toe pads


[7]. Also, capillary friction does not explain the scaling of friction with sliding speed and normal load. If present at all, capillary friction only plays a minor role in tree frog attachment.

### d Suction

Suction has been suggested early to explain tree frog attachment


[8]. In particular, the volume enclosed between the dimples found on the apical nanopillar surface


[1,7,9,10] and the substrate might give rise to suction (


[9]; Fig. SI.5). Importantly, the dimensions of the dimples could be distorted because of deeper AFM indentations in the centre than at the edges of the nanopillars due to a spatial variation in stiffness. In TEM measurements, shrinkage could distort the dimple shape.

By enlarging the enclosed volume, the pressure *P*_in_ in the enclosed region decreases relative to the environmental pressure *P*_env_. A pressure difference ∆*P* acting across the dimple surface *A* results in adhesion (Fig. SI.5, inset;


[11–13]):

| (SI.14) | $F_{\perp\text{,suc}}=\left(P_{\text{env}}-P_{\text{in}}\right) A=\Delta P A$ |
| --- | --- |

Typically, biological suckers are flexible, have a concave shape and a smooth rim for more effective sealing (e.g. by vdW interactions), and rely on muscles to change the size of the enclosed volume


[13].

In tree frogs, the secreted mucus might improve the sealing of the enclosed volume


[14] and the maximally achievable pressure difference of 1 bar (= 100 mN mm^-2^) would be sufficient to explain the measured adhesion of these animals. However, Emerson & Diehl


[12] found that the attachment of *Smilisca phaeota* to a vertical glass plate is not affected by a change of the environmental pressure *P*_env_ of ca. 30 mN mm^-2^ (i.e. 0.32 bar). Schuberg


[15] also described that *Hyla arborea* is able to climb in a partial vacuum. Further studies with experimental control of the environmental pressure are required to conclude on the role of suction in tree frogs. Because of the surface-normal orientation of suction forces, we do not expect a direct contribution of suction to the friction of the pads.

### e Kendall peeling model

At the onset of peeling of a thin, linearly elastic film (width *b*, thickness *h*, elastic modulus *E*, fracture energy per unit area *Δγ*) at a load *F*_L_ under an angle *θ*_L_ to the substrate over a distance *Δc* (Fig. SI.6), elastic energy (i.e. stretching of the film), potential energy (i.e. movement of the point of force application), and fracture energy (i.e. energy required to fracture a unit interfacial area) are in balance. A balance of fracture, potential and elastic energy yields


[16]:

| (SI.15) | $-\Delta\gamma b \Delta\text{c} +F_{\text{L}}\left(1-\cos\theta_{\text{L}}\right) \Delta\text{c}+ \frac{F_{\text{L}}^{2} \Delta\text{c}}{2 b E h}=0$ |
| --- | --- |

Rewriting Equation SI.15, one can compute the normal (adhesive) peeling force component:

| (SI.16) | $F_{\text{L},\perp}=\sin\theta b h E\left(\cos\theta-1+\sqrt{1-2 \cos\theta+\cos^{2} \theta+2 \frac{\Delta\gamma}{h E}}\right)$ |
| --- | --- |

The frictional component is calculated analogously by replacing sin *θ* with cos *θ*.

The fracture energy *∆γ* depends on peeling speed


[16] as well as adhesion


[17]. Measurement of *F*_⊥_ at *θ*_L_ = 90° allows an approximation of *∆γ* with Equation SI.15 for an inextensible film, because then *∆γ* = *F*_⊥_*/ b*. Based on data by Barnes et al.


[18], we obtain *∆γ* = [0*.*65 mN mm^−2^ · *π* (1*.*5 mm)^2^] */* [*π* (1*.*5 mm)^2^]^-0.5^ = 1*.*74 N m^−1^ for an assumed pad diameter *d*_p_ = 3 mm [average pad width *b*≈ (*π* *d*_p_^2^)^-0.5^].

Further, we estimate *∆γ* by fitting Equation SI.16 (*E* = 15 kPa, *h* = 0.5 mm) to peel off forces measured by Barnes et al.


[18] and Endlein et al. (


[19]; Fig. SI.7). The fitted curve represents measured data well (SE = 0*.*01 mN) and results in ∆γ = 1*.*24 N m^−1^. The peel forces for an inextensible and an elastic film deviate from each other by more than 5% at *θ*_L_ *<*16°, as to be expected


[16]. The finite peel off force components predicted by elastic theory at load angles approaching zero are more realistic than the continuously increasing forces in inextensible peeling


[19]. At *θ*_L_ *<*40°, no force data with controlled peeling angle are available as reference. The complex material properties (e.g. anisotropic or viscoelastic stiffness) of toe pads might prohibit the application of Kendall peeling theory at such low angles.

### f Johnson-Kendall-Roberts model

Johnson, Kendall & Roberts


[20] extended the Hertz model


[21] and implemented the effect of adhesion on the contact between two spherical objects. In the JKR-model, the work of adhesion *W*_⊥_, a system-specific quantity describing the energy per unit area required to irreversibly separate two objects


[20], of a sphere with radius *R* relates to the maximal adhesion *F*_⊥_ (i.e. pull off force) as:

| (SI.17) | $W_{\perp}=-\frac{2}{3}\frac{F_{\perp}}{\pi R}$ |
| --- | --- |

Using this model, Barnes et al.


[3] measured *W*_⊥_ ≈ 0*.*02–0*.*11 N m^−1^. Further, they reported a positive scaling of *W*_⊥_ with indentation depth and a negative scaling with pad size.

However, the JKR model is only valid for elastic objects and for small indentations compared to the sphere size


[22]. In contrast, tree frogs’ toe pads are potentially viscoelastic and presumably experience large deformations during contact. Further work is required to examine to what extent the JKR-model captures the contact mechanics of tree frogs’ toe pads accurately.

# References

[1.] Scholz I, Barnes WJP, Smith JM, Baumgartner W. Ultrastructure and physical properties of an adhesive surface, the toe pad epithelium of the tree frog, Litoria caerulea White. Journal of Experimental Biology. 2009;212:155–162.

[2.] Kitchener A, Vincent JF. Composite theory and the effect of water on the stiffness of horn keratin. Journal of Material Science. 1987;22:1385–1389.

[3.] Barnes WJP, Goodwyn PJP, Nokhbatolfoghahai M, Gorb SN. Elastic modulus of tree frog adhesive toe pads. Journal of Comparative Physiology A. Springer; 2011;197(10):969–978.

[4.] Vincent JFV, Wegst UGK. Design and mechanical properties of insect cuticle. Arthropod Structure & Development. 2004;33:187–199.

[5.] Smith JM, Barnes WJP, Downie JR, Ruxton GD. Adhesion and allometry from metamorphosis to maturation in hylid tree frogs: a sticky problem. Journal of Zoology. 2006;270:372–383.

[6.] Butt H-J, Barnes WJP, Campo A del, Kappl M, Schönfeld F. Capillary forces between soft, elastic spheres. Soft Matter. 2010;6:5930–5936.

[7.] Federle W, Barnes WJP, Baumgartner W, Drechsler P, Smith JM. Wet but not slippery: boundary friction in tree frog adhesive toe pads. Journal of The Royal Society Interface. The Royal Society; 2006;3(10):689–697.

[8.] Mohnike O. Ueber das Vermögen verschiedener Säugethiere sich mittels des atmosphärischen Druckes an glatten, mehr oder weniger senkrechten Flächen festhalten und aufwärts bewegen zu können. Zeitschrift für wissenschaftliche Zoologie. 1879;32:388–406.

[9.] Barnes WJP, Baum M, Peisker H, Gorb SN. Comparative Cryo-SEM and AFM Studies of Hylid and Rhacophorid Tree Frog Toe Pads. Journal of Morphology. 2013;274:1384–1396.

[10.] Ernst VV. The digital pads of the tree frog, Hyla cinerea. I. The epidermis. Tissue and Cell. 1973;5(1):83–96.

[11.] Ditsche P, Summers AP. Aquatic versus terrestrial attachment: Water makes a difference. Beilstein Journal of Nanotechnology. Beilstein-Institut; 2014;5(1):2424–2439.

[12.] Emerson SB, Diehl D. Toe pad morphology and mechanisms of sticking in frogs. Biological Journal of the Linnean Society. 1980;13:199–216.

[13.] Gorb SN. Biological attachment devices: exploring nature’s diversity for biomimetics. Philosophical Transactions of the Royal Society A. 2008;366:1557–1574.

[14.] Leydig F. Ueber Organe eines sechsten Sinnes. E. Blochmann & Sohn; 1868.

[15.] Schuberg A. Über den Bau und die Funktion der Haftapparate des Laubfrosches. Arbeiten aus dem Zoologisch-Zootomischen Institut in Würzburg [Internet]. 1891;10(1):57–119. Available from: https://archive.org/details/arbeitenausdemzo10semp

[16.] Kendall K. Thin-film peeling-the elastic term. Journal of Physics D: Applied Physics. 1975;8:1449–1452.

[17.] Pesika NS, Tian Y, Zhao B, Rosenberg K, Zeng H, McGuiggan JN P. Israelachvili, et al. Peel zone model of tape peeling based on the gecko adhesive system. Santa Barbara, USA: Department of Chemical Engineering, University of California Santa Barbara; 2006.

[18.] Barnes WJP, Pearman J, Platter J. Application of peeling theory to tree frog adhesion, a biological system with biomimetic implications. E-Newsletters for Science and Technology, Published by European Academy of Sciences (EAS). 2008;1(1):1–2.

[19.] Endlein T, Ji A, Samuel D, Yao N, Wang Z, Barnes WJP, et al. Sticking like sticky tape: tree frogs use friction forces to enhance attachment on overhanging surfaces. Journal of The Royal Society Interface [Internet]. 2012;10(80):1–11. Available from: http://dx.doi.org/10.1098/rsif.2012.0838

[20.] Johnson KL, Kendall K, Roberts AD. Surface Energy and the Contact of Elastic Solids. Proceedings of the Royal Society of London. Series A, Mathematical and Physical Sciences [Internet]. 1971;324(1558):301–313. Available from: http://www.jstor.org/stable/78058

[21.] Kendall K. Molecular Adhesion and Its Applications - The Sticky Universe. New York, Boston, Dordrecht, London, Moscow: Kluwer Academic Publishers; 2004.

[22.] Popov V. Contact Mechanics and Friction: Physical Principles and Applications. Heidelberg, Dordrecht, London, New York: Springer; 2010.

[23.] Barnes WJP, Oines C, Smith JM. Whole animal measurements of shear and adhesive forces in adult tree frogs: insights into underlying mechanisms of adhesion obtained from studying the effects of size and scale. Journal of Comparative Physiology A. Springer; 2006;192(11):1179–1191.

[24.] Smith JM, Barnes WJP, Downie JR, Ruxton GD. Structural correlates of increased adhesive efficiency with adult size in the toe pads of hylid tree frogs. Journal of Comparative Physiology A. Springer; 2006;192(11):1193–1204.

# Figures

**Figure SI.1:** Geometrical parameters of our model of the ventral surface of a tree frog’s toe pad. (A) Calculation of the projected surface area of a single epidermal cell (*A*_c_, dark) and the surrounding channel (light). (B) Calculation of the projected surface area of a single nanopillar (medium), the surrounding channel (light) and the dimple (dark). *d*_c_ cell diameter, *a*_c_ cell edge length, *w*_c_ channel width, α*_c_* opening angle, *A*_c_ apical surface area, *d*_n_ nanopillar diameter, *w*_n_ nanopillar channel width, ∆*r* distance between dimple and nanopillar edge. CH channel, DI dimple, NP nanopillar.

**Figure SI.2:** Measured maximum pad tenacity *σ*_⊥_ in various hylids (circles: redrawn from Fig. 3D in


[23]; crosses: redrawn from Fig. 1D in


[24]). The scaling of *σ*_⊥_ with snout-vent-length *ℓ*_SV_ is modelled linearly (dotted) and as exponential growth decay (dashed).

**Figure SI.3:** Function principle of the rotation table experiment. A frog is placed on a platform that rotates (around a horizontal axis) into an overhanging position and the angles of initial slipping (A, *α*_||_) and falling (B, *α*_⊥_) of the animal are recorded. Based on vector decomposition and measurement of body mass, the maximum adhesion (*F*_⊥_) and friction (*F_||_*) as well as the coefficient of static friction (*µ_||_*) are computed.

**Figure SI.4:** Model of capillary adhesion between a sphere and a deformable flat plate (*R*_2_ = ∞). *d*_g_ gap width, *F*_⊥,cap_ capillary adhesion, *R*= *R*_1_ radius of sphere, *R*_mer_, *R*_azi_ meridional and azimuthal radius of meniscus curvature, *γ* mucus surface tension, *φ*_1_, *φ*_2_ sphere-liquid and substrate-liquid contact angle.

**Figure SI.5:** Schematic representation of the suction between ventral toe pad epidermis (green) and substrate (grey). Suction forces *F*_⊥,suc_ might arise at a normal load *F*_⊥_*_,_*_L_ from a difference between environmental pressure *P*_env_ and the pressure *P*_in_ in the volumes enclosed between the ‘dimples’ on the apical surfaces of the nanopillars and the substrate (inset; red).

**Figure SI.6:** Peeling of a thin film


(16). *E* elastic modulus, *h* film thickness, *F*_L_ Load, *Δc* peeling distance, *θ*_L_ peeling angle.

**Figure SI.7:** Peeling force components of single toe pads of *Litoria caerulea* (*E* = 15 kPa, *d*_p_ = 3 mm, *h* = 0.5 mm, *A*_p_ = π (*d*_p_ / 2)^2^, b = *A*_p_^-0.5^; crosses: redrawn from Fig. 1B in


[18]; circles: recomputed from Fig. 6 in


[19]) for uncontrolled peeling angles. Non-linear total least squares regression of the surface-normal (dashed) peeling force component of an elastic film (dark) to the data by Barnes et al.


[18] gives Δγ = 1.24 Nm^−1^ ± 0.003 Nm^−1^ (95% CI, SE = 0.01 mN). Parallel peeling forces (dashed-dotted) and the peel off force of a stiff film (light) are shown for comparison. Adhesion deducted from rotation table measurements (dotted) serves as reference (Fig. SI.2).
